# Experimental and numerical studies of the impact breakage of granite with high ejection velocities

**DOI:** 10.1371/journal.pone.0266241

**Published:** 2022-04-07

**Authors:** Penglin Zhang, Zhijun Wu, Jinglai Sun, Yang Liu, Zhaofei Chu

**Affiliations:** 1 Chengdu Engineering Corporation limited, Chengdu, China; 2 School of Civil Engineering, Wuhan University, Wuhan, China; 3 Beijing Municipal Engineering Research Institute, Beijing, China; University of Science and Technology Beijing, CHINA

## Abstract

The impact-induced fragmentation of rock is widely and frequently encountered when natural hazards occur in mountainous areas. This type of fragmentation is an important and complex natural process that should be described. In this study, laboratory impact tests under different impact velocities were first conducted using a novel gas-driven rock impact apparatus. The three-dimensional digital image correlation (3D DIC) technique was used to monitor the dynamic fragmentation process upon impact. Then, coupled 3D finite-discrete element method (FDEM) numerical simulations were performed to numerically investigate the energy and damage evolutions and fragmentation characteristics of the sample under different impact velocities. The laboratory test results show that as the impact velocity increases, the failure pattern of the rock sample gradually changes from shear failure to splitting failure, and the fragmentation intensity increases obviously. The strain localization area gradually increases as the impact velocity increases and as the location gradually deviates away from the impacting face. In the numerical simulation, the proposed model is validated by quasi-static uniaxial compression tests and impact tests. The numerical simulations clearly show the progressive fracture process of the samples, which agrees well with the experimental observations. The evolutions of energy and damage variables were also derived based on the simulation results, which are markedly affected by the impact velocity. The fragment size distributions based on mass and number can be well fitted using a generalized extreme value law. Finally, the distribution of the fragment flying velocity and angle are analyzed.

## 1. Introduction

Impact-induced fragmentation of rock blocks is widely observed in incidents of natural hazards such as rockfalls, sturzstroms, rockslides and rock avalanches [1–4], which in turn markedly influences the motion trajectory of rock blocks [5, 6]. Rock fragmentation, which features high kinetic energy and undefined motion trajectory, poses critical threats to human lives, infrastructure and lifeline facilities [7–12]. However, impact-induced fragmentation of rock blocks is a complex breakage process that includes impact dynamics, fracture mechanics and rock mechanics [13–15]. Therefore, to mitigate the hazard of impact-induced flying fragments, it is essential to investigate the dynamic fracturing process and fragmentation characteristics.

Extensive efforts have been made to understand the dynamic fragmentation mechanism of rock blocks under impacts [4, 16–19]. As the most direct and reliable method among those methods used in previous studies, laboratory experiments have been widely conducted in attempts to describe the fracturing and fragmentation mechanism. Laboratory tests on brittle materials [20–22] have shown that the number of fragments increases with increasing impact energy, while fragment size gradually decreases. Giacomini et al. [2] conducted in situ free fall tests and found that the impact angle played an important role in the fragmentation of foliated rocks, and the influence of the impacting energy tended to be of the second order. Hou et al. [23] conducted an experimental study of the fragmentation characteristics of brittle rocks and found that as impact energy increases and hammer size decreases, the frequency distribution curve becomes narrower, and the cumulative frequency distribution curve gradually moves toward the left. To describe the fragmentation behaviors of rock under impact loading in more detail, several scholars have investigated the characteristic fragment distribution of rock. Hogan et al. [24] developed a three-parameter generalized extreme value distribution to describe characteristic fragment sizes. By fitting a model to 448 sets of screening fragment size data from blasting and crushed rock [25], bicomponent distributions were found to generally create a better fit, and Swebrec is the best single component function in all zones, producing errors that are comparable to the best bicomponents in the coarse and central zones. In addition, Li et al. [26] explored the dynamic fragmentation of granite at strain rates of 40-150/s and proposed a novel energy-based fragmentation model to describe cylindrical samples compacted by single direction impact.

In addition to experimental approaches, with the development of computational techniques, numerical methods are an efficient alternative to investigate impact-induced dynamic fragmentation, which can generally be categorized into three types: continuum-based numerical methods, discontinuum-based methods and hybrid methods. Among these methods, the most widely used approach is the discrete element method (DEM) [27–29], which is well suited to model multibody dynamics. DEM simulations have contributed to our fundamental understanding of rock dynamic fragmentation [30–34], and several DEM simulation results [31, 32, 35] have shown that the normal component of impact velocity is the primary factor that affects the breakage intensity of agglomerates and fragmentation distribution. Based on DEM simulation results, fragmentation only occurs locally at the impact zone, but no radial cracks occur [14, 36]. Shen et al. [17] found that as the impact loading rates increased, the fragmentation intensity and damage ratio gradually increased, and the fragment number exhibited a power law dependence on the impact loading rate. Zhao et al. [6] studied rock fragmentation at various slope angles using the DEM. Numerical results indicated that steep slopes, which resulted in higher impact strain rates, induced more efficient fragmentation than gentle slopes, and the fragment size decreased with increasing impact strain rate. Zhao et al. [37] also investigated the dynamic fragmentation characteristics of jointed rock blocks and found that the orientation and distribution of the block joints are the main factors affecting the size and shape of large fragments. They also found that the cumulative size distribution can be well fitted by Weibull’s distribution function, with gentle and steep curvatures with fine and coarse size ranges, respectively. However, although the DEM has been favored by many researchers in rock fragmentation studies due to its advantage of being able to simulate fracturing, this method is time-consuming and requires an error-prone calibration of micro-to-macro material properties, increasing computational costs, especially in large-scale computing. Among the continuum-based methods, the numerical erosion technique is widely used to simulate the dynamic failure process of materials [38–40]. However, the erosion technique lacks physical meaning, and substantial element deletion breaches the conservation of mass [41]. Therefore, to more realistically simulate the dynamic failure process, more advanced methods based on FEM have been proposed within the framework of the partition of unity (PU) to overcome the difficulty of FEM, which can be applied to dynamic crack propagation problems with an appropriate enrichment function and level set algorithm [42–44]. However, for extreme breaking failure under high-speed loads, adopting appropriate enrichment functions remains challenging, and the level set description is still not adequately robust [45, 46]. Alternatively, an effective method to avoid distortion or coincidence of elements with crack geometries is to use meshless methods [47, 48]. Based on the smooth particle hydrodynamics method (SPH), Rabczuk et al. [49] simulated concrete fragmentation under explosive loading. However, the difficulties of essential boundary conditions, tensile inability and zero energy modes are unavoidably encountered when using this type of method [50]. For example, continuum-based methods have difficulties describing the sticking, slipping and separation between fragments due to the continuum-based assumption. Recently, the combined finite-discrete element method (FDEM) has become popular and has been widely applied to simulate the impact-induced fracture and fragmentation process of rock material [51, 52] due to its inherent continuum-discontinuum characteristics. In the FDEM [53–55] regarding the material as a number of interactive discrete elements with general shapes and sizes, the key feature of rock impact-induced failure process simulation involves the easy transition from continuum to discontinuum via deformation, fracture and fragmentation. Using the contact algorithm, the cohesive crack model can be described accurately.

In this paper, a gas-driven rock impact apparatus is developed and used to conduct laboratory rock impact tests. The dynamic crack growth, strain localization and final failure pattern of granite specimens under different impact velocities are investigated in detail using the 3D-DIC technique. Then, a coupled 3D FDEM model is created in Abaqus software to simulate the energy and damage evolutions and fragmentation characteristics of the granite specimens in the impact tests and compared with the laboratory impact tests. The progressive fracture process; the evolution of energy and damage; and fragmentation and its size distribution and distribution of the fragment flying velocity and angle are described by the FDEM simulation, which facilitates understanding the dynamic fragmentation process and fragmentation characteristics of rock.

## 2. Experimental and numerical methodology

### 2.1 Specimen preparation and test equipment

The granite specimens investigated in this study were sourced from Changsha City in Hunan Province, China. Cylindrical specimens 50 mm in diameter and 100 mm in height were produced according to the International Society for Rock Mechanics (ISRM)-suggested methods [56]. To reduce the discretization of test results caused by specimen inhomogeneity, all specimens were drilled from the same rock block, and specimens with similar P-wave velocities were selected for testing. The basic mechanical properties of the specimens were described from unconfined compression tests. The density (*ρ*) was approximately 2.63 g/cm^3^, and the P-wave velocity was 4957 m/s. The Young’s modulus (*E*) was 40.29 GPa, Poisson’s ratio (*υ*) was 0.25 and the uniaxial compressive strength (UCS) was approximately 102.66 MPa. To enhance the contrast ratio of the rock surface for the digital image correlation analysis, all the granite specimens were treated by spraying speckle. During spraying, white speckle was first sprayed throughout the observation area, and then, black speckle was evenly sprayed until the black speckles were uniformly distributed on the specimen surface.

**Fig 1:**
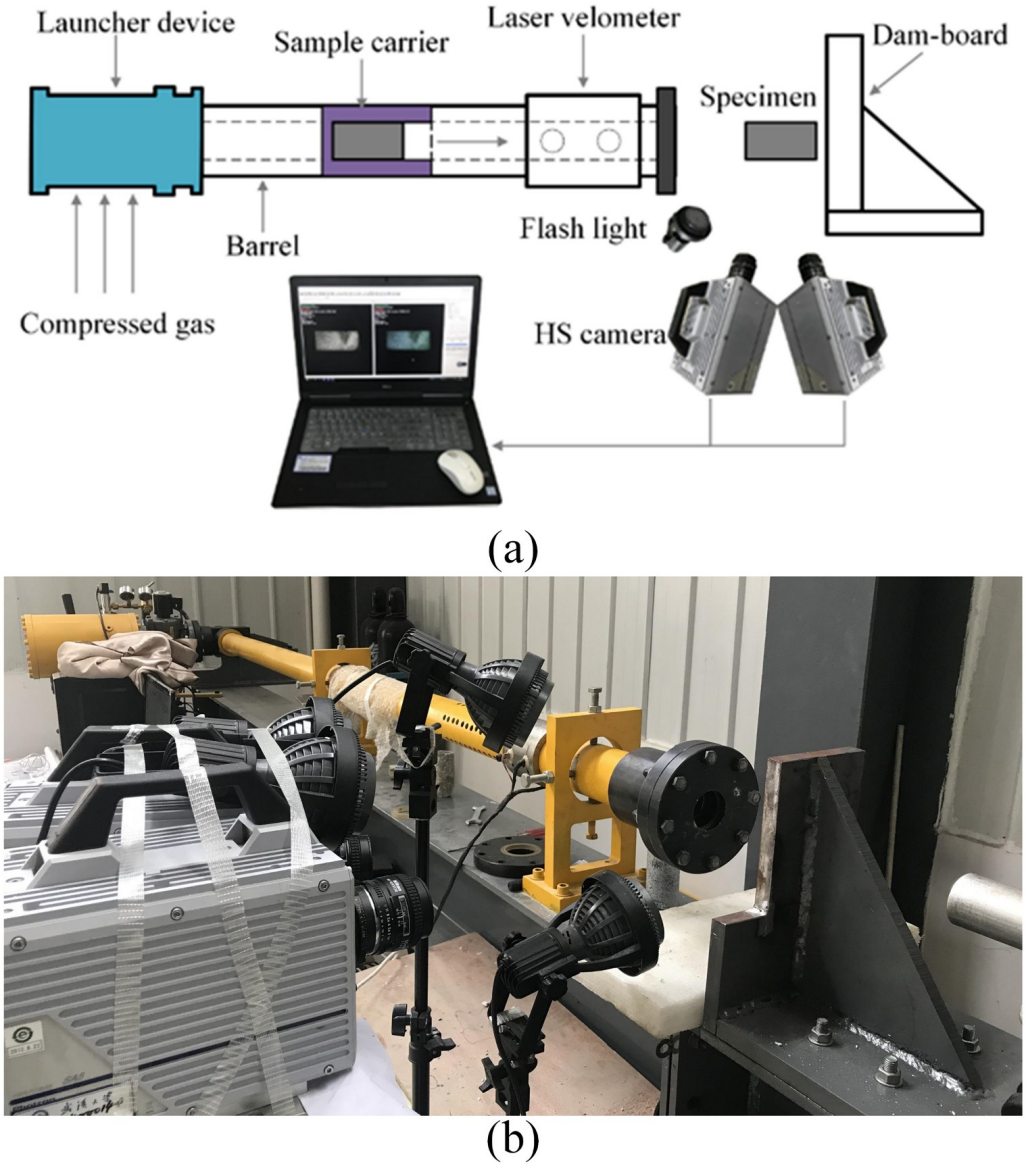
The compressed gas-driven impact system. (a) schematic diagram; (b) test device.

A gas-driven rock impact apparatus was developed for the impact test, which consists of five main components (the launcher component, specimen carrier, laser velometer, damboard and high-speed shooting system), as shown in Fig 1a and 1b.

To investigate the influences of impact velocities on the rock failure behaviors and fragmentation characteristics, impact tests with different impact velocities were conducted on the rock specimens by adjusting the compressed gas. During the impact tests, the error under different impact numbers of the same impact velocity was controlled to be within ±0.3 m/s. Therefore, to better understand the mechanism of rock breakage under high impact velocities, two synchronous high-speed cameras were used to monitor and record each impact. A 3D-DIC technique was also used to analyze variations in the 3D strain fields of the sample surface [57–59]. The coordinates of the 3D-DIC method first had to be calibrated, which required deducing the 3D coordinates from the projected plane coordinates obtained from the two cameras. By moving, tilting and rotating the calibration board, twelve image pairs of calibration boards were captured to calibrate the position of the two cameras. The calibration board used was a plastic board (90 mm × 72 mm) with a black base and 25 × 22 white speckles. The calibration was adequate for measurement when the standard deviation of the residuals calculated by the software (GOM Correlate Professional) was less than 0.1. After calibration was completed, the image pairs were recorded at the ideal frame and resolution by the two synchronized cameras, and the cropping adjustment function in the software could recognize the actual resolution and apply a modified calibration to the speckle images. The frame rate was set to 35000 fps; thus, the time interval between two neighboring images was approximately 27 μs. Finally, the strain fields were obtained by calculating valuable images with GOM Correlate Professional software.

### 2.2 Numerical model

In the FDEM model, the rock mass was idealized as a collection of elastic bulk elements, and their interactions between the rock mass boundaries were described by the zero-thickness cohesive elements. Before failure, the constitutive relation of the elastic element and the stiffness of the cohesive elements were used to control the continuous behaviors of the rock material. However, the discontinuous failure process was described by the constitutive properties of the zero-thickness cohesive element. The zero-thick cohesive element were completely damaged and then deleted from the model when it met the failure criterion, resulting in the generation of cracking. Crack propagation occurred with the continuous deletion of the zero-thick cohesive element. In addition, the interaction between the elastic bulk elements generated after the deletion of the zero-thick cohesive element could be achieved by defining the contact.

**Fig 2:**
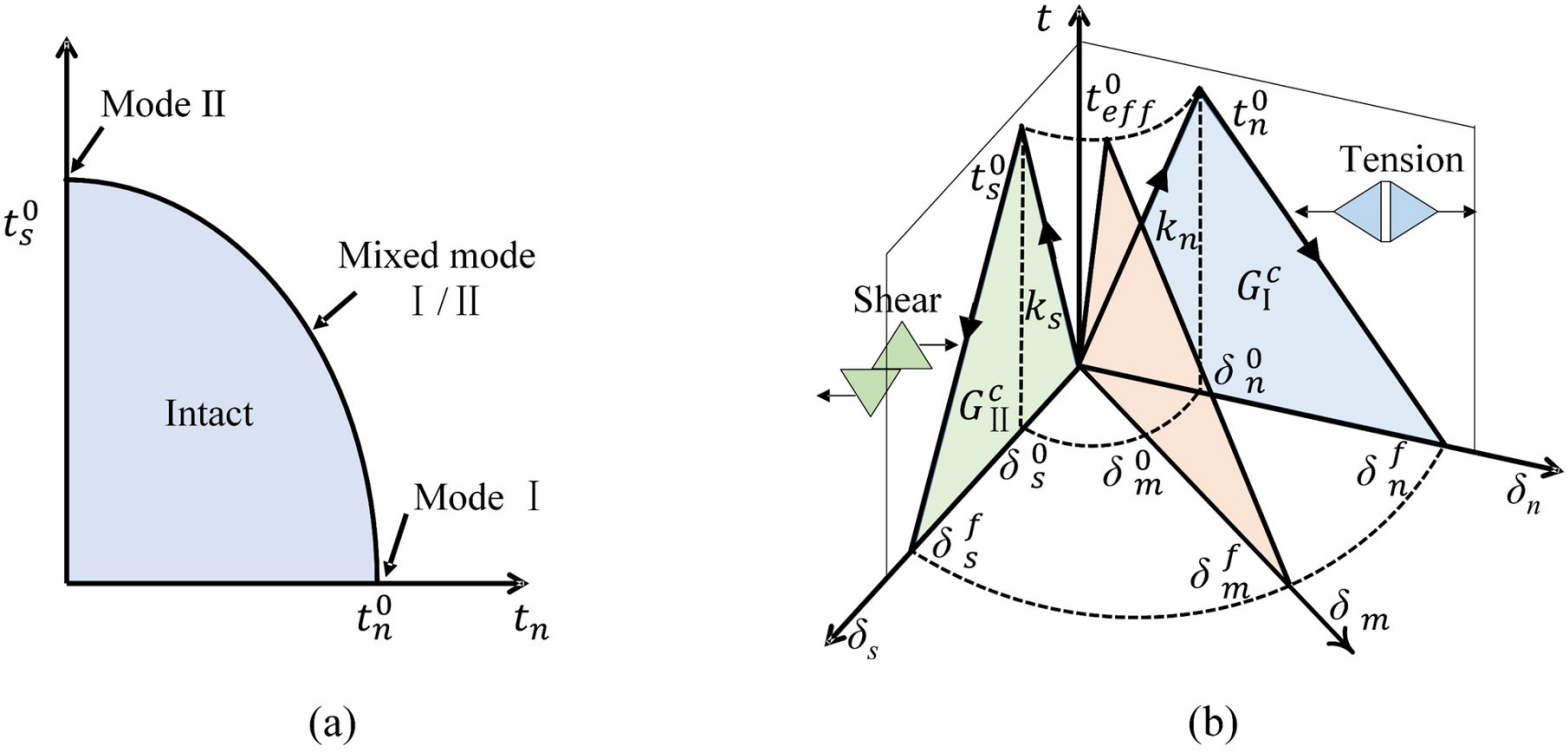
Constitutive model of cohesive elements. (a) mixed-mode damage initiation criterion; (b) mixed-mode linear traction-separation behavior.

The constitutive response of zero-thickness cohesive elements could be directly defined by the traction-separation curve, which could be applied to simulate the cracking behavior at the solid element boundary. In this study, the mechanical behaviors of cohesive elements were defined by a mixed-mode bilinear traction-separation law, as shown Fig 2. In the constitutive model, the traction in each direction increased linearly with the increase in the relative displacement before damage initiation. When the traction of the cohesive element reached the maximum nominal traction criterion, damage began to be generated. The damage initiation criterion was described by the quadratic nominal stress law (Fig 2a) as follows:

$${\left\{\frac{\langle t_{n}\rangle }{t_{n}^{0}}\right\}}^{2}+{\left\{\frac{t_{s}}{t_{s}^{0}}\right\}}^{2}=1$$ (1)

where 〈〉 is the Macaulay bracket, indicating that the cohesive elements could not show compression (negative normal traction); *t*_*n*_ and *t*_*s*_ denote the nominal (mode І) and shear (mode II) traction, respectively; and $t_{n}^{0}$ and $t_{s}^{0}$ are the nominal and shear traction when the damage initiates, respectively. As shown in Fig 2b, the curve in the plane formed by the *t*-axis and the *δ*_*n*_-axis stands for tensile behavior; the curve in the plane formed by the *t*-axis and the *δ*_*s*_-axis stands for share behavior; the curve in the plane formed by the *t*-axis and the *δ*_*m*_-axis stands for tensile-shear mixed behavior. The damage evolution is described by the scalar damage variable *D*, which is a function of the effective relative traction *t*_*eff*_ and separation *δ*_*m*_, considering the coupled effect of both the normal and shear directions:

$$t_{eff}=\sqrt{{\langle t_{n}\rangle }^{2}+t_{s}^{2}}$$ (2)

$$\delta_{m}=\sqrt{{\langle \delta_{n}\rangle }^{2}+\delta_{s}^{2}}$$ (3)

where *δ*_*n*_ and *δ*_*s*_ are the normal and shear separations, respectively. The scalar damage variable *D* initially has a value of 0. If the damage is modeled, *D* monotonically evolves from 0 to 1 until the cohesive elements complete failure, as follows:

$$D=\{\begin{array}{ll} \frac{\delta_{m}^{f}(\delta_{m}^{max}-\delta_{m}^{0})}{\delta_{m}^{max}(\delta_{m}^{f}-\delta_{m}^{0})}, & \delta_{m}^{max}\geq \delta_{m}^{0}\\ 0, & \delta_{m}^{max}<\delta_{m}^{0} \end{array}$$ (4)

where,$\delta_{m}^{0}$, $\delta_{m}^{f}$ and $\delta_{m}^{\text{max}}$ are the effective separation at damage initiation, the effective separation at complete failure and the maximum value of the effective separation attained during the loading history, respectively.

The evolution of *D* is also accompanied by the degradation of the normal and shear stiffness as well as the normal and shear traction of the cohesive elements until complete failure. The constitutive law can be expressed as follows:

$$t_{n}=\{\begin{array}{ll} (1-D)k_{n}\delta_{n}, & \delta_{n}\geq 0\\ k_{n}\delta_{n}, & \delta_{n}<0 \end{array}$$ (5)

$$t_{s}=(1-D)k_{s}\delta_{s}$$ (6)

The mixed-mode complete failure is governed by the following power law failure criteria:

$${\left(\frac{G_{I}}{G_{IC}}\right)}^{2}+{\left(\frac{G_{II}}{G_{IIC}}\right)}^{2}=1$$ (7)

where *G*_*I*_ and *G*_*II*_ are the dissipated energies in pure mode I and mode II, respectively; and *G*_*IC*_ and *G*_*IIC*_ are the fracture energies in pure mode I and mode II, respectively. The cohesive element will be complete failure and deleted to show the local failure as the criterion is met.

After the cohesive elements are deleted, the penalty contact scheme is used to define the contact behaviors between the newly created adjacent surfaces. The penalty stiffness can also be set as a constant or adjusted automatically to allow for a little penetration. The contact stress-relative displacement relation is described as follows:

$$p=\{\begin{array}{ll} 0, & \delta_{n}\geq 0\\ -p_{n}\delta_{n}, & \delta_{n}<0 \end{array}$$ (8)

$$\tau =p_{s}\delta_{s}$$ (9)

where *p*, *p*_*n*_ and *δ*_*n*_ are the contact stress, penalty stiffness and relative replacement in the normal direction, respectively; and *τ*, *p*_*s*_ and *δ*_*s*_ are the corresponding quantities in the shear direction.

The critical shear stress is calculated using Coulomb’s law as follows:

$$\tau_{cri}=\mu p$$ (10)

where *μ* describes the friction coefficient of the contacting surfaces. In this paper, *μ* = 0.3.

## 3. Experimental results and analyses

### 3.1 Failure pattern

**Fig 3:**
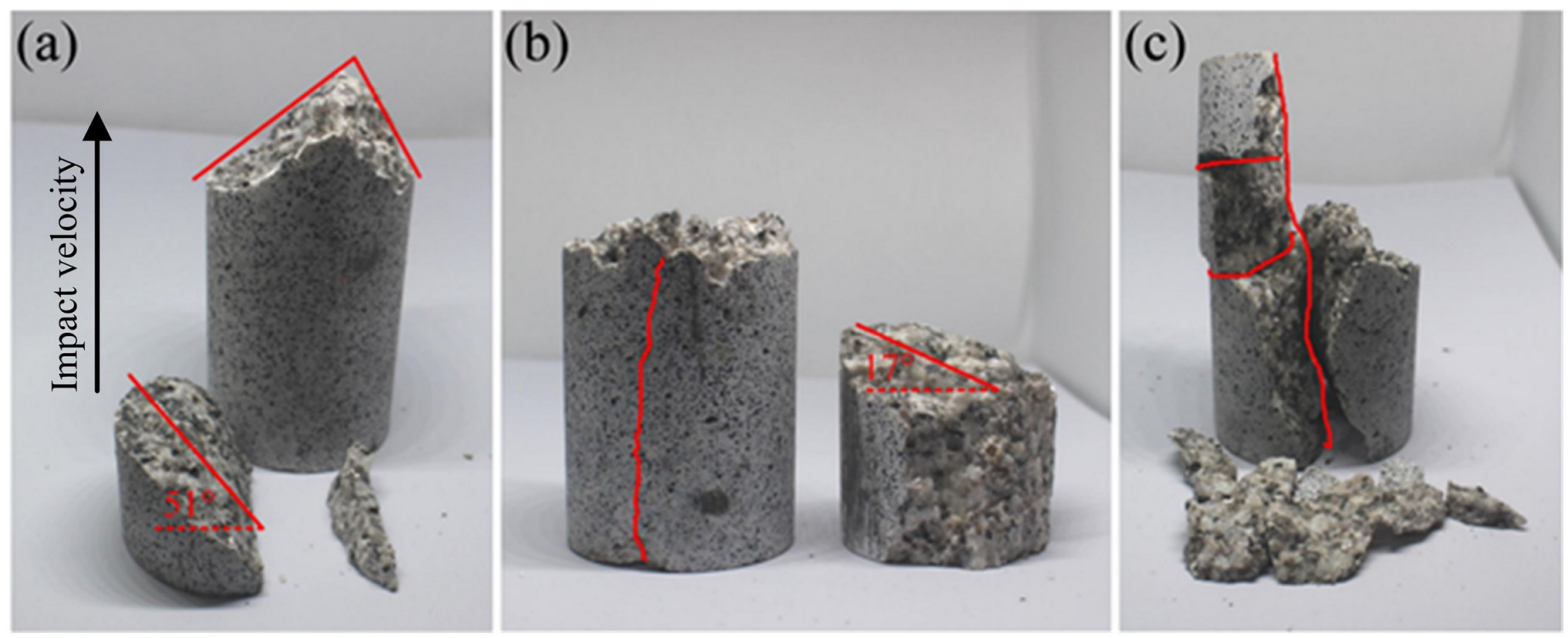
Failure patterns of rock specimens subjected to different impact velocities. (a) 20.0 m/s, (b) 25.0 m/s, and (c) 30.0 m/s.

The failure pattern of a rock specimen after impact loading depends on the loading rate [60–62]. Examples of different failure patterns for rock specimens under different impact velocities are shown in Fig 3, and results show that as the impact velocity gradually increases from 20.0 to 30.0 m/s, the fragmentation intensity continues to increase. At a velocity of 20.0 m/s, no obvious cracks are found in the remaining part of the specimen away from the impacting face, and a fracture plane occurs near the impacting face. When the impact velocity increases to 25.0 m/s, an obvious vertical crack appears in the remaining part of the specimen away from the impacting face. As the impact velocity increases more to 30.0 m/s, the remaining part of the specimen away from the impacting face is directly split into several blocks along the impact direction. In addition, as the impact velocity increases from 20.0 to 25.0 m/s, the angle of the fracture plane gradually decreases from 51° to 17°, and the location of the fracture plane gradually moves away from the impacting face. This result is probably due to the low impact-induced stress at low impact velocities, and the impact-induced stress is gradually attenuated when it propagates inside the rock specimen. Thus, the part near the impacting face is directly broken by the impact-induced stress, and no obvious cracks are generated in the remaining part of the specimen away from the impacting face. As the impact velocity increases, the impact-induced stress reaching the part away from the impacting face also increases, resulting in the fracture moving away from the impacting face. A vertical crack then appears in the remaining part of the specimen away from the impacting face. When the impact velocity increases more to 30.0 m/s, the impact-induced stress far exceeds the dynamic stress of the specimen, and the location of the specimen near the impacting face is directly squeezed and broken. Finally, the part away from the impacting face directly fails by splitting, and the fragmentation intensity of the whole rock specimen obviously increases.

### 3.2 Strain measurement and fracture characteristics

The 3D-DIC technique can be used to quantitatively characterize the strain response of the surface of a rock specimen in the impact process. For moving specimens, 3D-DIC can directly measure the strain through speckle movement on the specimen surface, which can effectively capture the spatial strain field of the rock specimen surface at the moment of impact breakage. In this study, the processes of crack initiation and propagation and the subsequent macroscopic failure of the rock specimen during impact are monitored and recorded by two high-speed cameras. The image at 0 μs is used as the reference image, and a zone-of-interest (ZOI) of 576 × 320 pixels is selected for correlation calculation. The size of one pixel is equivalent to 0.15 mm of the specimen, and the 3D-DIC technique can capture only half the area of the sample surface due to the limitation of the high-speed camera, which would cause the capture of cracks to have a certain randomness.

**Fig 4:**
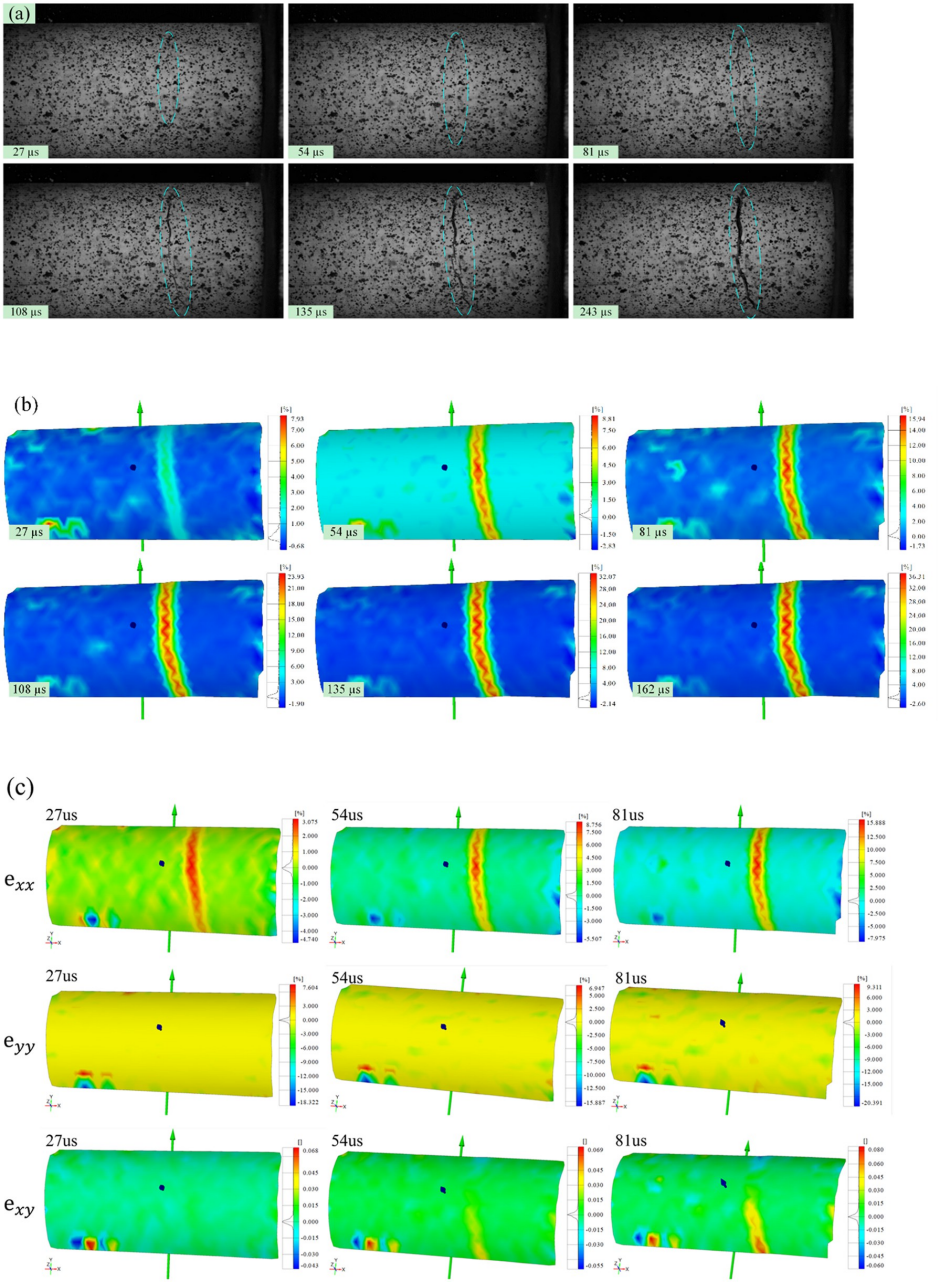
Images of the evolution of visible cracks and strain fields in different directions at a velocity of 20.0 m/s (the right face is the impacting face). (a) the high-speed images; (b) the maximum principal strain; (c) the axial (e_xx_), vertical (e_yy_) and shear (e_xy_) strain.

**Fig 5:**
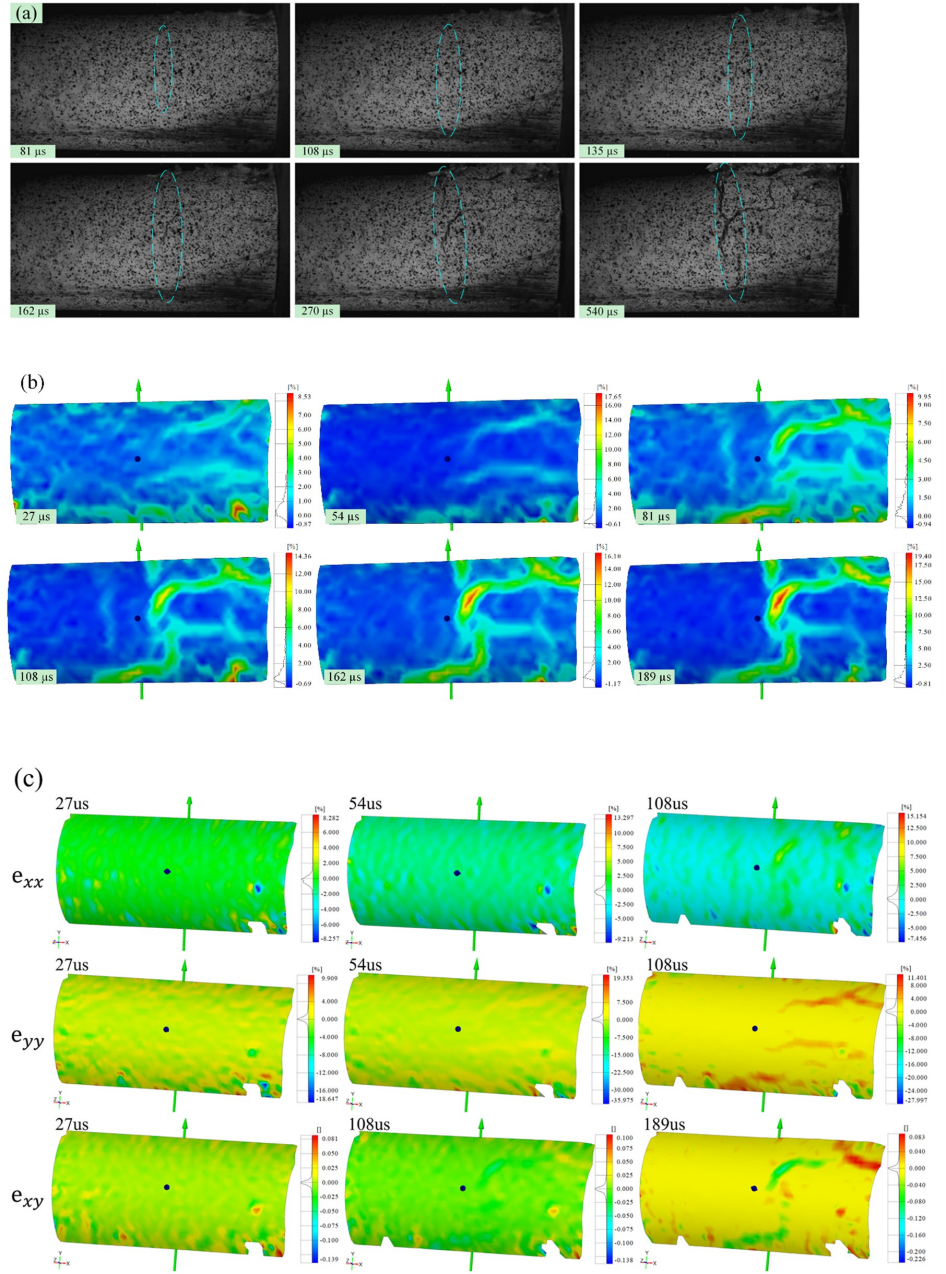
Images of the evolution of visible cracks and strain fields in different directions at a velocity of 25.0 m/s (the right face is the impacting face). (a) the high-speed images; (b) the maximum principal strain; (c) the axial (e_xx_), vertical (e_yy_) and shear (e_xy_) strain.

**Fig 6:**
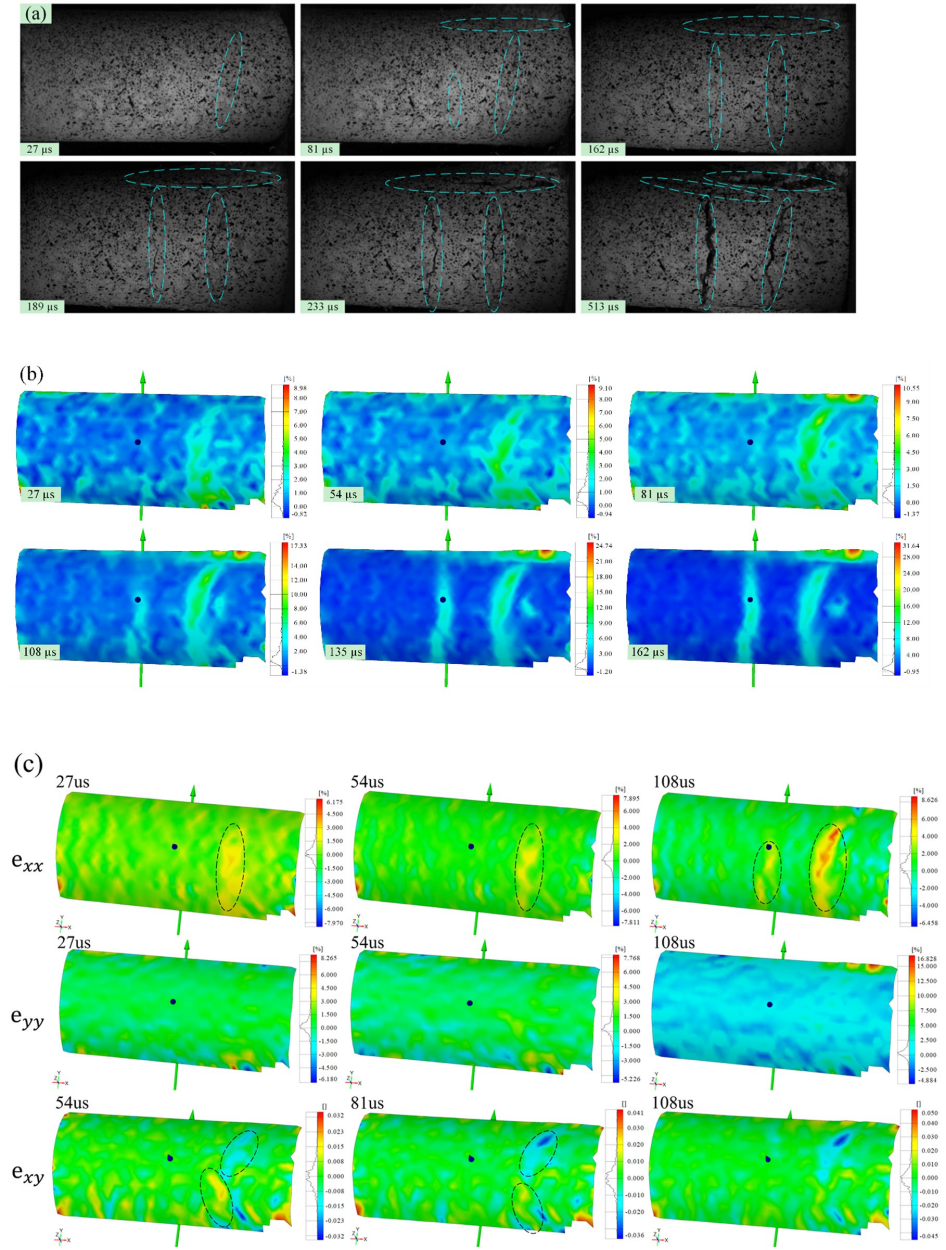
Images of the evolution of visible cracks and strain fields in different directions at a velocity of 30.0 m/s (the right face is the impacting face). (a) the high-speed images; (b) the maximum principal strain; (c) the axial (e_xx_), vertical (e_yy_) and shear (e_xy_) strain.

High-speed images of the evolution of visible cracks at the moment of impact-induced breakage under velocities of 20.0, 25.0, and 30.0 m/s are shown in Figs 4a, 5a and 6a, respectively. Figs 4b, 5b and 6b show the strain fields under impact velocities of 20.0, 25.0 and 30.0 m/s in the maximum principal strain directions at different stages. Figs 4c, 5c and 6c show the strain fields under impact velocities of 20.0, 25.0 and 30.0 m/s in the axial (e_xx_), vertical (e_yy_) and shear (e_xy_) directions at different stages. Under an impact velocity of 20.0 m/s (see Fig 4), a crack appears at 27 μs along with obvious strains in the maximum principal strain and axial directions. The crack initiates at the location farthest from the impacting face and then rapidly expands from the location at the bottom of the fracture plane to both sides until it reaches the impacting face. In addition, an obvious shear strain localization is shown at 54 μs, which indicates that the fracture plane may be formed due to shear failure during impact. When the impact velocity increases to 25.0 m/s (see Fig 5), the strain localization area gradually increases with increasing impact velocity. In the axial direction, an obvious strain change can be observed, and the area of compressive-shear strain (the strain value is positive) is larger than the tensile strain (the strain value is negative) before 54 μs. However, in the vertical direction, the strain localization area is almost tensile strain. At 108 μs, an obvious strain localization area appears in both the axial and vertical directions. Furthermore, at 108 μs, strain localization appears in the shear direction, which is more obvious at 189 μs. These results indicate that the sample is markedly compressed in the axial direction and expanded in the radial direction during the impact process. Compared with the crack initiation figures (Fig 5a), most visible cracks are caused by tensile-shear failure. As the impact velocity increases more to 30.0 m/s (see Fig 6), cracks parallel to the impact direction appear. In addition, two cracks perpendicular to the impact direction appear successively after the impact and also appear earlier when they are closer to the impacting face. In the axial direction, the tensile strain area gradually increases over time, while the compressive strain area decreases significantly, and the difference between the two is most prominent at 108 μs. In the vertical direction, both the tensile strain and its area gradually increase over time, and the tensile strain area is significantly larger than that under a velocity of 25.0 m/s. In the shear direction, strain localization can be observed at the location away from the impacting face. In addition, shear strain localization also appears in the crack propagation tip, which indicates that the 3D-DIC technique can accurately predict the crack propagation direction through changes in the strain field. Finally, according to the results of the strain field and previous failure pattern, the failure pattern appears to gradually change from shear failure to splitting failure with increasing impact velocity.

## 4. Numerical simulation results

### 4.1 Calibration of the numerical model

**Fig 7:**
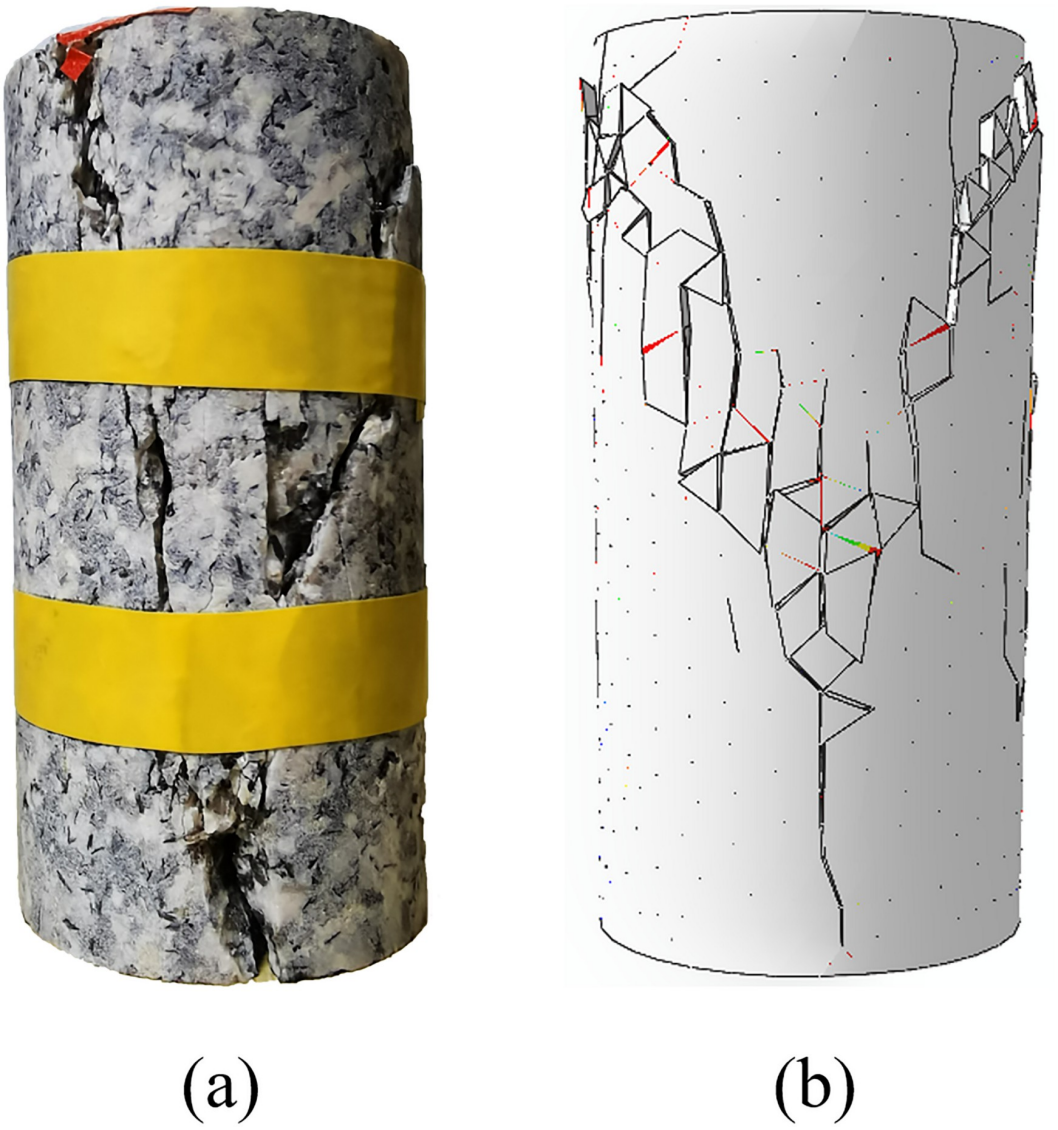
Uniaxial compressive test. (a) the failure mode of granite sample obtained from laboratory test; (b) the failure mode predicted by the numerical model.

**Fig 8:**
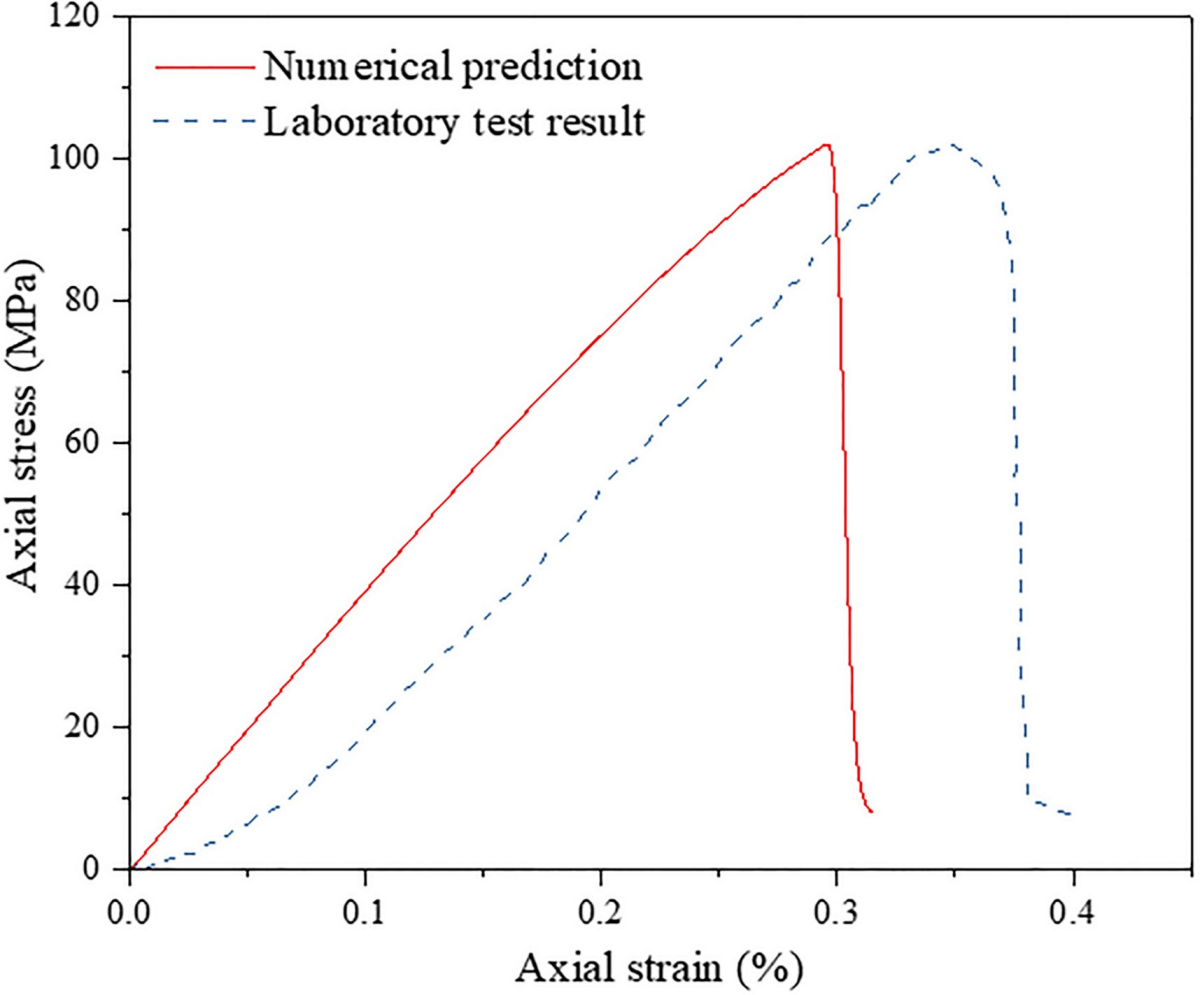
Comparison of the axial stress–strain curves of the granite sample between the laboratory test and numerical prediction.

In the numerical study, some micromechanical parameters of the numerical model cannot be directly obtained from laboratory tests, but can only be obtained through calibration. Cylindrical samples with a size of 50 mm × 100 mm are used. Two rigid platens are applied in this model, in which the upper platen is moving downward at a constant velocity of 2.5 mm/s to maintain a quasi-static loading state while the lower platen is fully fixed. The mean grain size of the granite sample observed from the laboratory is approximately 2 mm, which is smaller than the adopted size. However, if an actual mean grain size of 2 mm is adopted, more than 96460 solid elements and 187072 cohesive elements will be generated, which will inevitably increase computation time, especially for the 3D model used in this study. To compromise between modeling efficiency and accuracy, the numerical model is generated by irregular tetrahedral solid elements with a mean grain size of 4 mm as well as six-node zero-thick cohesive elements, corresponding to 14108 and 26674 elements. The solid element adopts linear elastic constitutive, which needs three parameters: density *ρ*, elastic modulus *E* and Poisson’s ratio *υ*. These three parameters are directly obtained from the laboratory tests (as shown Table 1). The cohesive constitutive needs six parameters: tensile strength $t_{n}^{0}$; shear strength $t_{s}^{0}$; mode І fracture energy *G*_*IC*_; mode II fracture energy *G*_*IIC*_; initial normal stiffness *k*_*n*_ and initial shear stiffness *k*_*s*_. Among them, *k*_*n*_ and *k*_*n*_ are usually estimated by empirical equation:

$$k_{n},k_{s}=\frac{\alpha E}{h_{mesh}}$$ (11)

where, *h*_*mesh*_ correspond to element mesh size, and *α* is a constant which needs to be determined. In this paper, when calculating the initial normal stiffness, the value of *α* is about 100, and when calculating the initial shear stiffness, the value of *α* is about 30. However, $t_{n}^{0}$, $t_{s}^{0}$, *G*_*IC*_ and *G*_*IIC*_ can’t be directly obtained from the laboratory tests. Therefore, firstly, the granite data in the reference [63] are selected as the initial value. Then, several simulations of uniaxial compression test are conducted. By comparing the stress-strain curves obtained by the simulations with the stress-strain curves obtained by the test, the values of these four parameters are continuously adjusted. After many trial-and-error calibration procedures, the simulation results after calibration are obtained, as shown Table 1, which agree with the results derived from the laboratory tests. The comparison of the macroscopic failure mode between the numerical tests and laboratory tests is shown in Fig 7, and the axial stress–strain curves predicted by the numerical simulation are shown in Fig 8. Note that some special aspects of rock behaviors, such as the closure of voids and pre-existing cracks, are not considered in the simulation, which causes axial stress–strain curves in the simulation to be slightly different from those of the laboratory tests, particularly where the initial compaction is not reflected in the numerical results. Generally, a good approximation of the macromechanical properties as well as the fracture behaviors can be measured in the numerical model. Results demonstrate that the calibrated micromechanical parameters, as shown in Table 2, are considered to be valid and can be used in the following numerical simulations.

**Table 1:** Comparisons of the basic mechanical parameters of the granite samples.

	Uniaxial compressive strength (MPa)	Young’s modulus (GPa)	Poisson’s ratio
Laboratory test result	102.66	40.29	0.25
Numerical test result	102.05	38.64	0.24

**Table 2:** Microparameters of the granite samples for the numerical model.

Microparameters	Values
Density, *ρ* (g/cm^3^)	2.63
Young’s modulus, *E* (GPa)	40.29
Poisson’s ratio *υ*	0.25
Tensile strength, $t_{n}^{0}$ (MPa)	11.2
Shear strength, $t_{s}^{0}$ (MPa)	45.5
Mode І fracture energy, *G*_*IIC*_ (N/mm)	0.134
Mode ІI fracture energy, *G*_*IIC*_ (N/mm)	0.47
Initial normal stiffness, *k*_*n*_ (MPa/mm)	1.0×10^6^
Initial shear stiffness, *k*_*s*_ (MPa/mm)	3.4×10^5^

**Fig 9:**
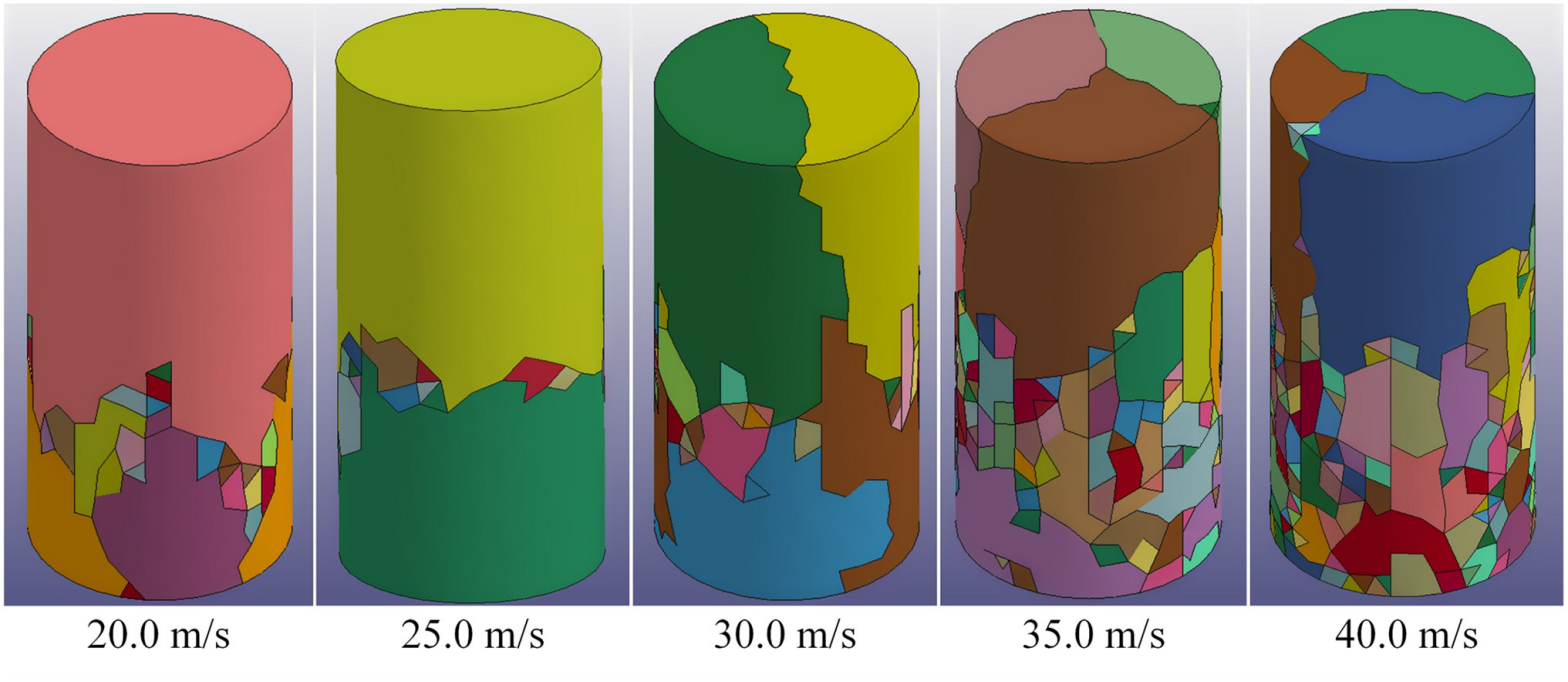
Numerical simulation results of granite samples under different impact velocities (the lower face is the impacting face).

To verify the effectiveness of this approach in simulating the micro/macromechanical properties of rocks, a numerical test of rock impact is conducted, and its results are compared with the laboratory test results. For comparison, the same impact velocities of 20.0, 25.0 and 30.0 m/s used in the laboratory tests are used in the numerical tests of rock impact. Fig 9 shows the fragmentation results of the sample under different impact velocities obtained from numerical simulations. When the impact velocity is 20.0 and 25.0 m/s, fragmentation occurs in the location near the impacting face, and several fragments are generated. When the impact velocity increases to 30.0 m/s, in addition to a number of fragments generated in the location near the impacting face, the remaining part of the sample away from the impacting face is directly split into two large fragments along the impact direction. The numerical test results agree with the results obtained from laboratory tests, as shown in Fig 3, and results show that the proposed method can accurately simulate the macromechanical properties and fragmentation characteristics of intact rock with the above calibrated micronumerical parameters. Based on this method, we continue to perform numerical simulations of rock impact at higher impact velocities, which may cause dangerous accidents in the laboratory. Fig 9 indicates that as the impact velocity increases, the fragmentation intensity of the sample gradually increases, and the remaining part of the sample away from the impacting face is directly split into three large fragments along the impact direction.

### 4.2 Energy and damage evolution during the progressive fracture process

**Fig 10:**
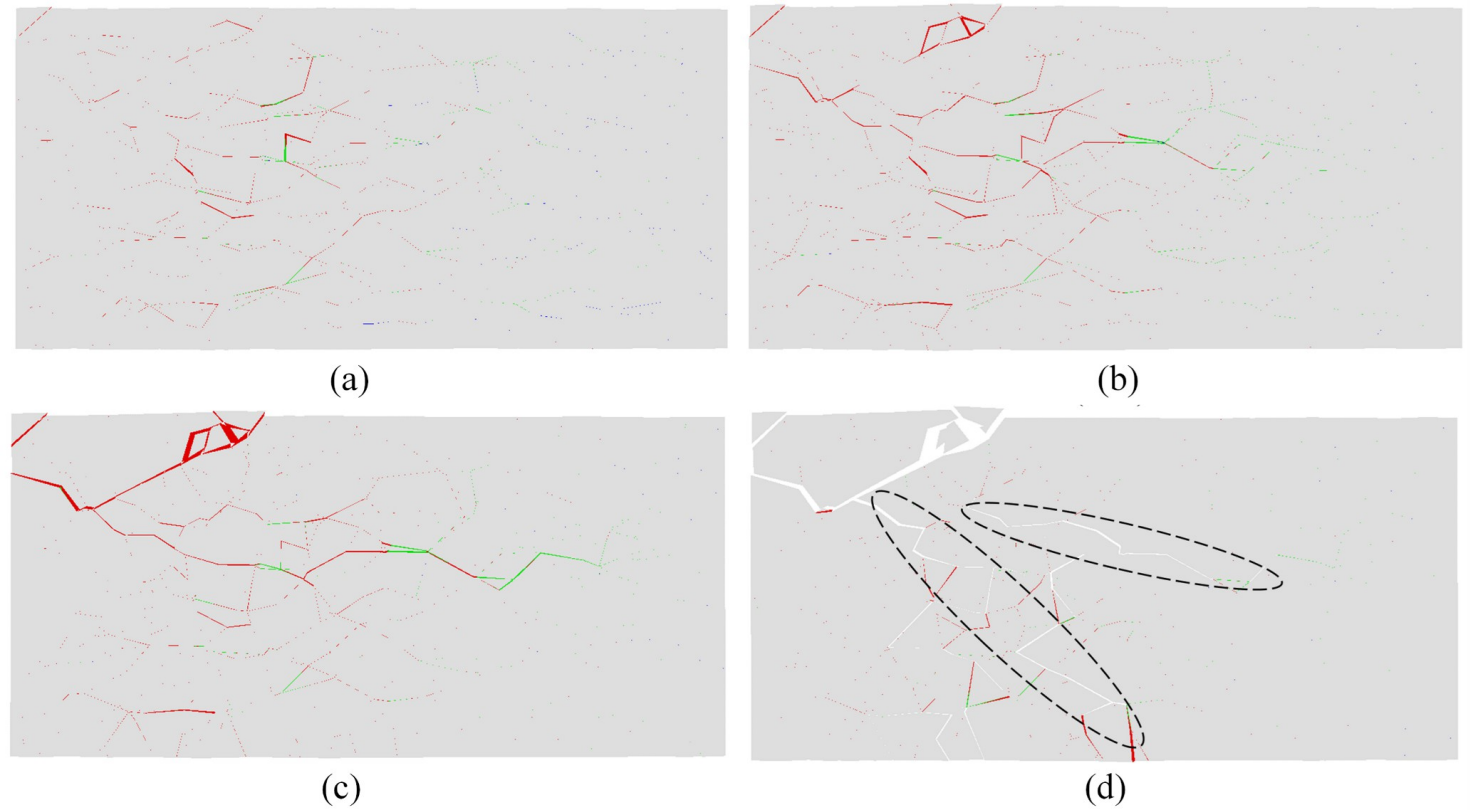
Progressive fracture process of the slice at a velocity of 20.0 m/s (the left face is the impacting face, and the red and green traces depict the cohesive element that has been deleted mainly due to shear failure and tensile failure, respectively): (a) at 0.04 ms; (b) at 0.08 ms; (c) at 0.12 ms; (d) at 0.12 ms, the deleted cohesive elements are not displayed.

**Fig 11:**
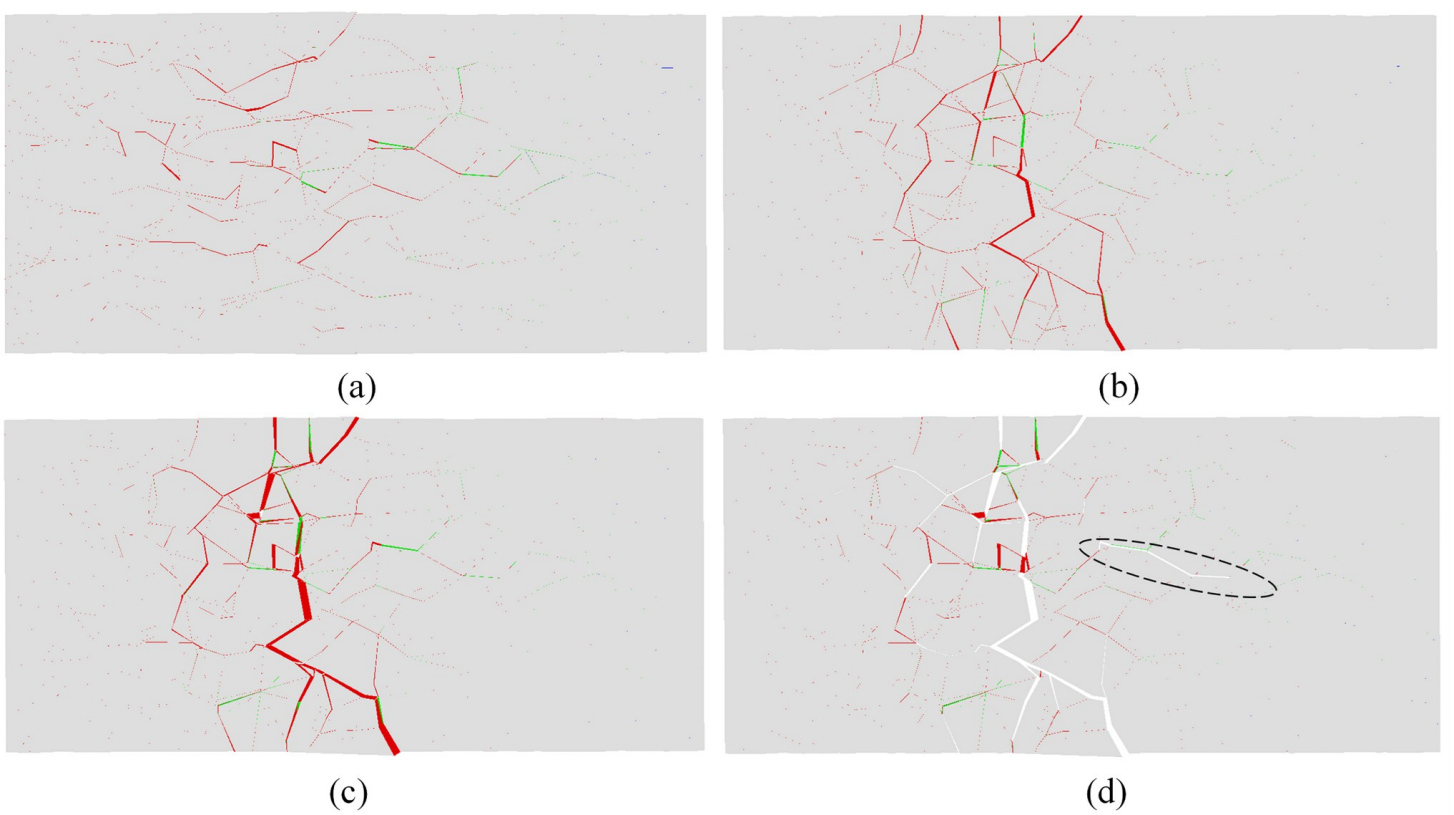
Progressive fracture process of the slice at a velocity of 25.0 m/s (the left face is the impacting face, and the red and green traces depict the cohesive element that has been deleted mainly due to shear failure and tensile failure, respectively): (a) at 0.1 ms; (b) at 0.2 ms; (c) at 0.25 ms; (d) at 0.25 ms, the deleted cohesive elements are not displayed.

**Fig 12:**
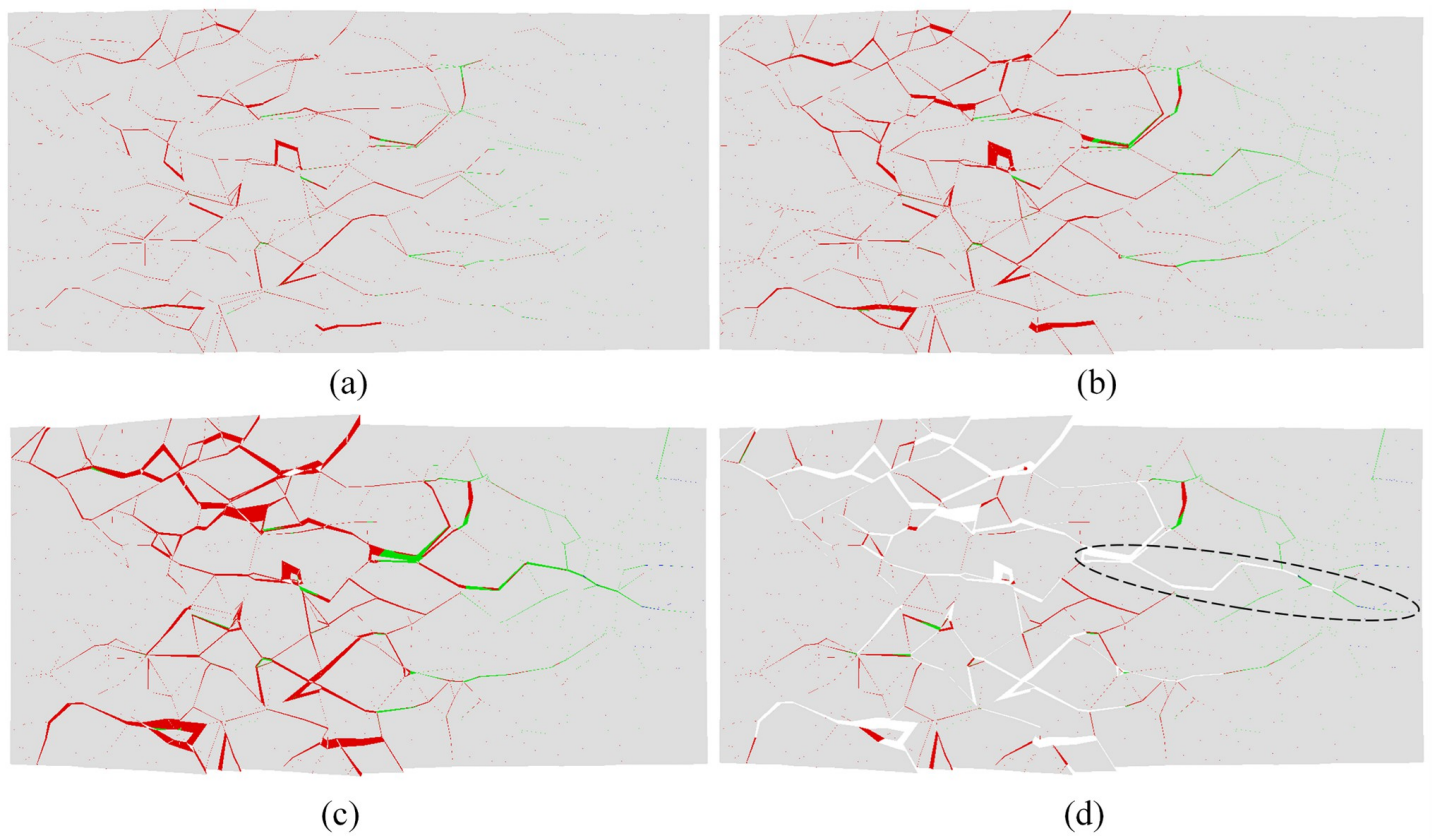
Progressive fracture process of the slice at a velocity of 30.0 m/s (the left face is the impacting face, and the red and green traces depict the cohesive element that has been deleted mainly due to shear failure and tensile failure, respectively): (a) at 0.06 ms; (b) at 0.09 ms; (c) at 0.15 ms; (d) at 0.15 ms, the deleted cohesive elements are not displayed.

Based on the proposed method described in Section 2.2, the progressive fracture process of the rock sample can be captured intuitively from the numerical simulation results. Fig 10 shows the progressive fracture process of the selected slice at a velocity of 20.0 m/s, in which the slice is the view of the central cross section in the x-y plane. The left end of the sample near the impacting face was directly crushed via shear, and additional shear cracks appeared at the location far away from the impacting face. In the part of the sample that is far away from the impacting face, a long tensile crack along the impact direction was generated and gradually closed over time; however, this phenomenon cannot be reproduced in the laboratory tests. When the impact velocity increases to 25.0 m/s, both the fragmentation intensity and the number of cracks increase, as shown in Fig 11. A number of shear cracks perpendicular to the impact direction were generated at 0.2 ms, and the tensile and shear cracks began to unite and gradually formed a fracture surface at 0.25 ms. A long tensile crack along the impact direction is also shown in the part of the sample far away from the impacting surface, which is in good agreement with that obtained from the laboratory tests (see Fig 3). As the impact velocity increases to 30.0 m/s, the number of damaged cohesive elements significantly increases at time 0.06 ms (as shown in Fig 12). With the deletion of the failed cohesive elements, a large number of cracks were generated, especially near the impacting face. In the part of the sample that is far away from the impacting face, a long tensile crack was generated and gradually penetrated from the middle to the back of the sample, which made the sample directly split into two fragments. According to these three figures, the part of the sample near the impacting face was squeezed at impact to produce radial expansion and shear failure, and as the impact velocity increased, the squeezed part fractured. Shear cracks were also generated near the impacting face at the moment of impact, and tensile cracks were formed far away from the impacting face during the rebound of the sample. Finally, macroscopic fracture surfaces were produced via the combination of tensile and shear cracks.

**Fig 13:**
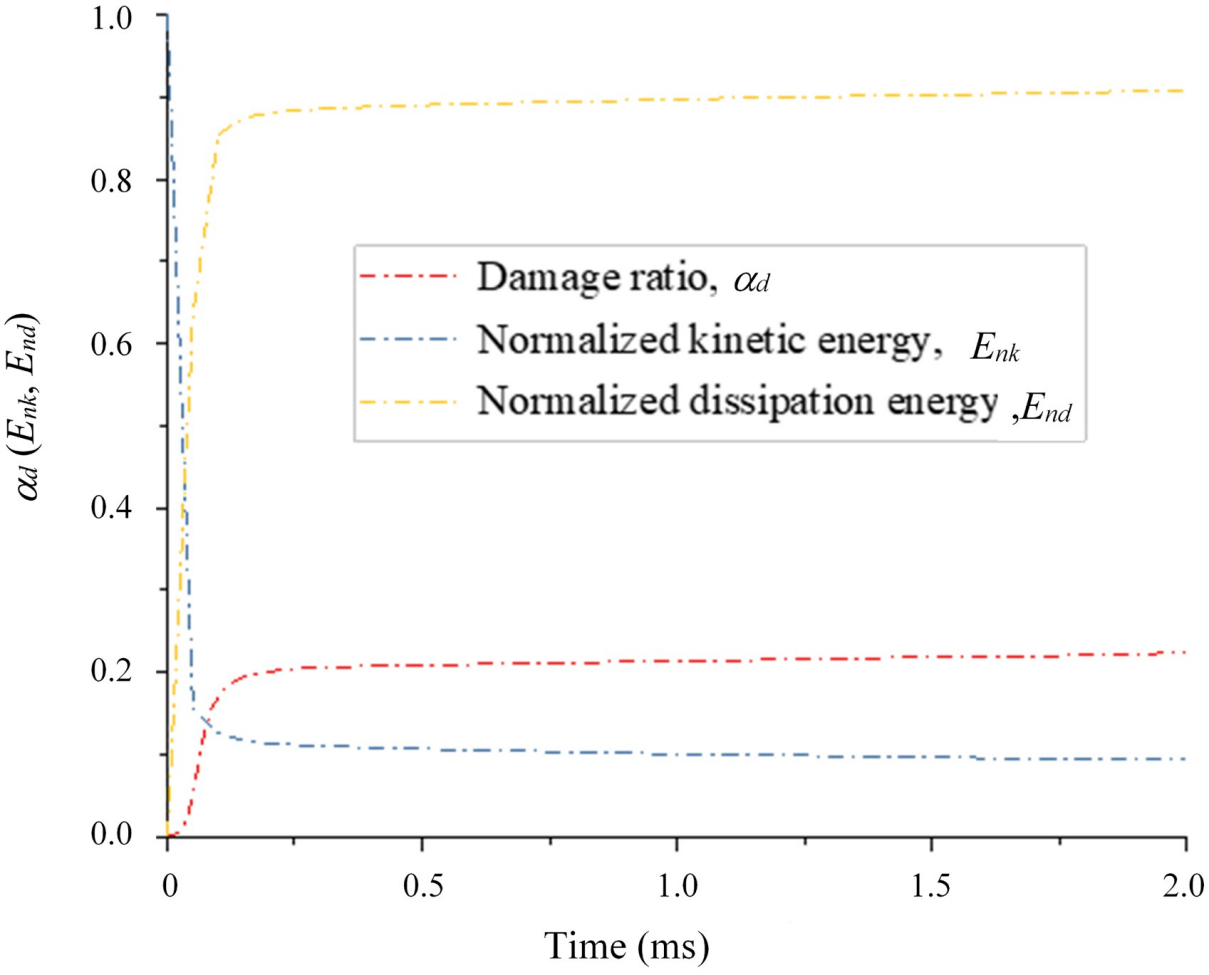
Evolution of the damage ratio (*α*_*d*_), normalized kinetic energy (*E*_*nk*_) and normalized dissipation energy (*E*_*nd*_) with time (*v*_*0*_ = 40 m/s).

**Fig 14:**
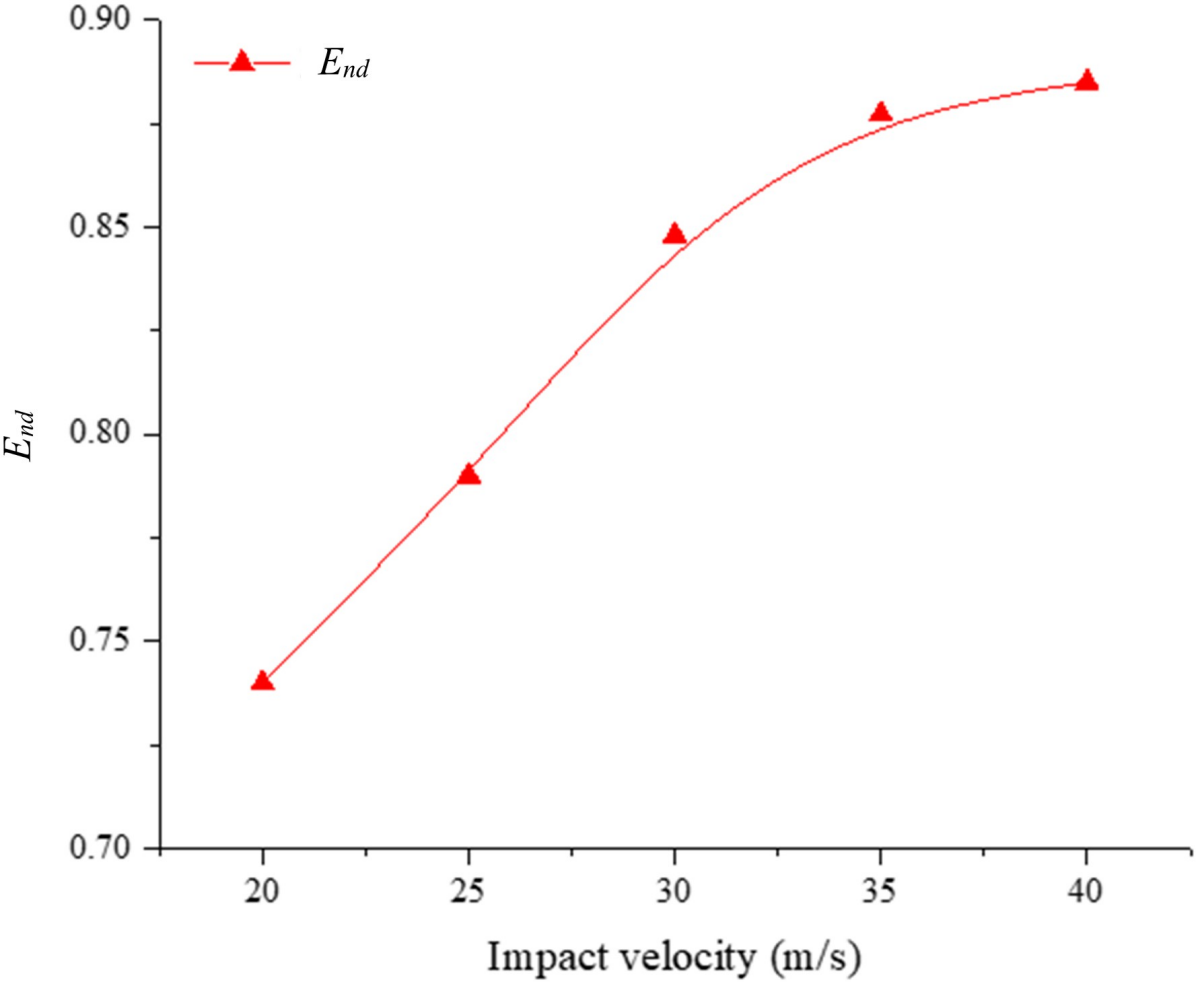
Variation in the energy dissipation (*E*_*nd*_) with the impact velocity.

As a key indicator of dynamic behavior, energy can be used to help analyze the energy consumption mechanism, damage and fragmentation transformation of rocks during impact. The damage ratio (*α*_*d*_) is the ratio of the number of failed cohesive elements to the initial number of cohesive elements, the normalized kinetic energy (*E*_*nk*_) is the ratio of the total kinetic energy of the fragments to the initial kinetic energy (*E*_*0*_), and the normalized dissipation energy (*E*_*nd*_) is the ratio of the dissipated energy to the initial kinetic energy. Fig 13 shows the evolution of the damage ratio, the normalized kinetic energy and the normalized dissipation energy over time for an impact velocity of 40.0 m/s. As shown in Fig 13, when the sample impacts the dam board, the damage ratio and normalized dissipated energy increase sharply up to their peak values at approximately 0.125 ms, and the normalized kinetic energy decreases dramatically. The sliding and collision of the fragments also yield a reduction in the normalized kinetic energy, while the damage ratio remains almost unchanged. Fig 14 shows the relationship between the normalized dissipated energy and the impact velocity, which indicates that the normalized dissipated energy gradually increases with increasing impact velocity. As the impact velocity increases, the increase in the normalized dissipated energy gradually slows down. Especially when the impact velocity increases to 40.0 m/s, the normalized dissipated energy only increases by 0.015.

**Fig 15:**
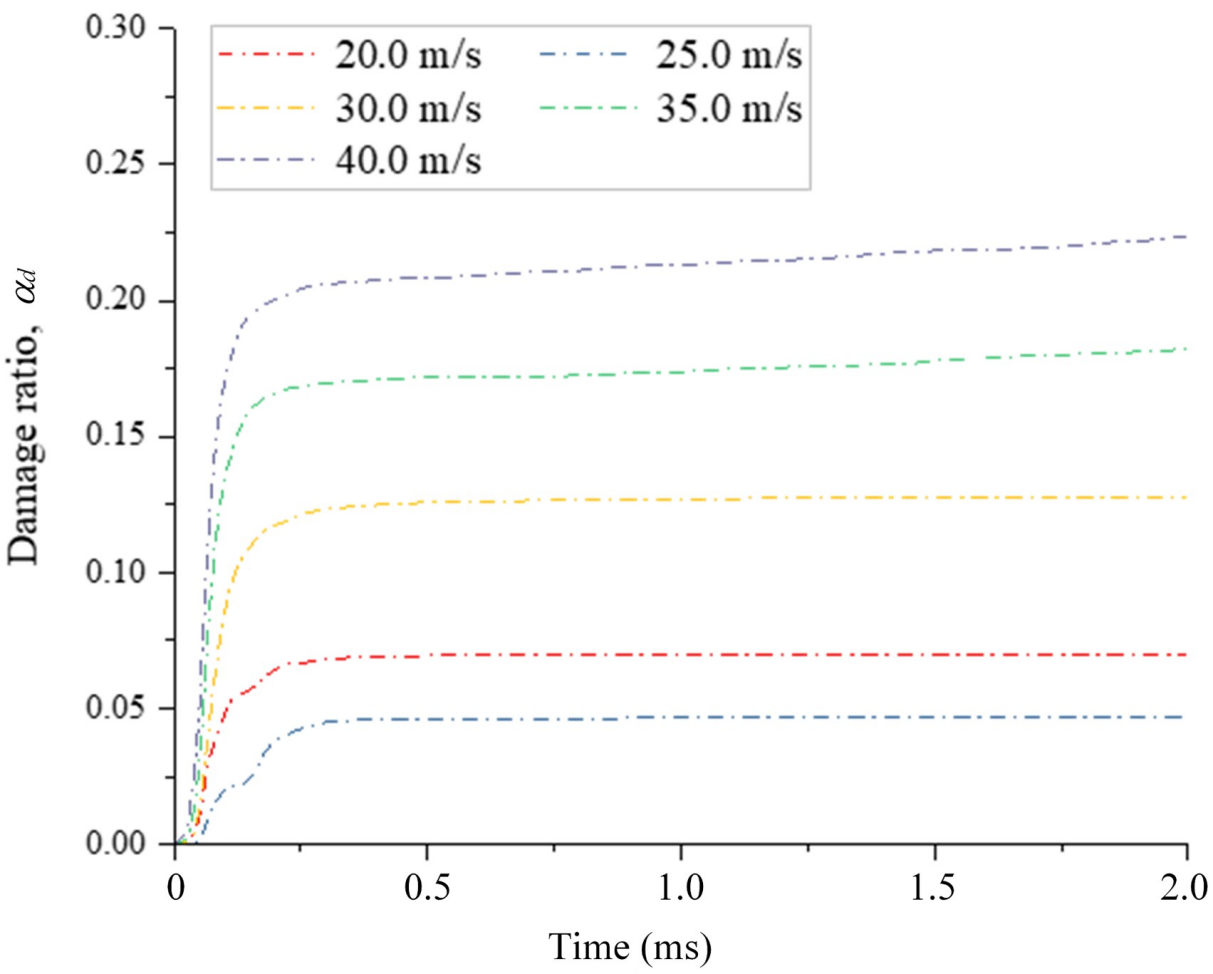
Evolution of the damage ratio (*α*_*d*_) with time under different impact velocities.

**Fig 16:**
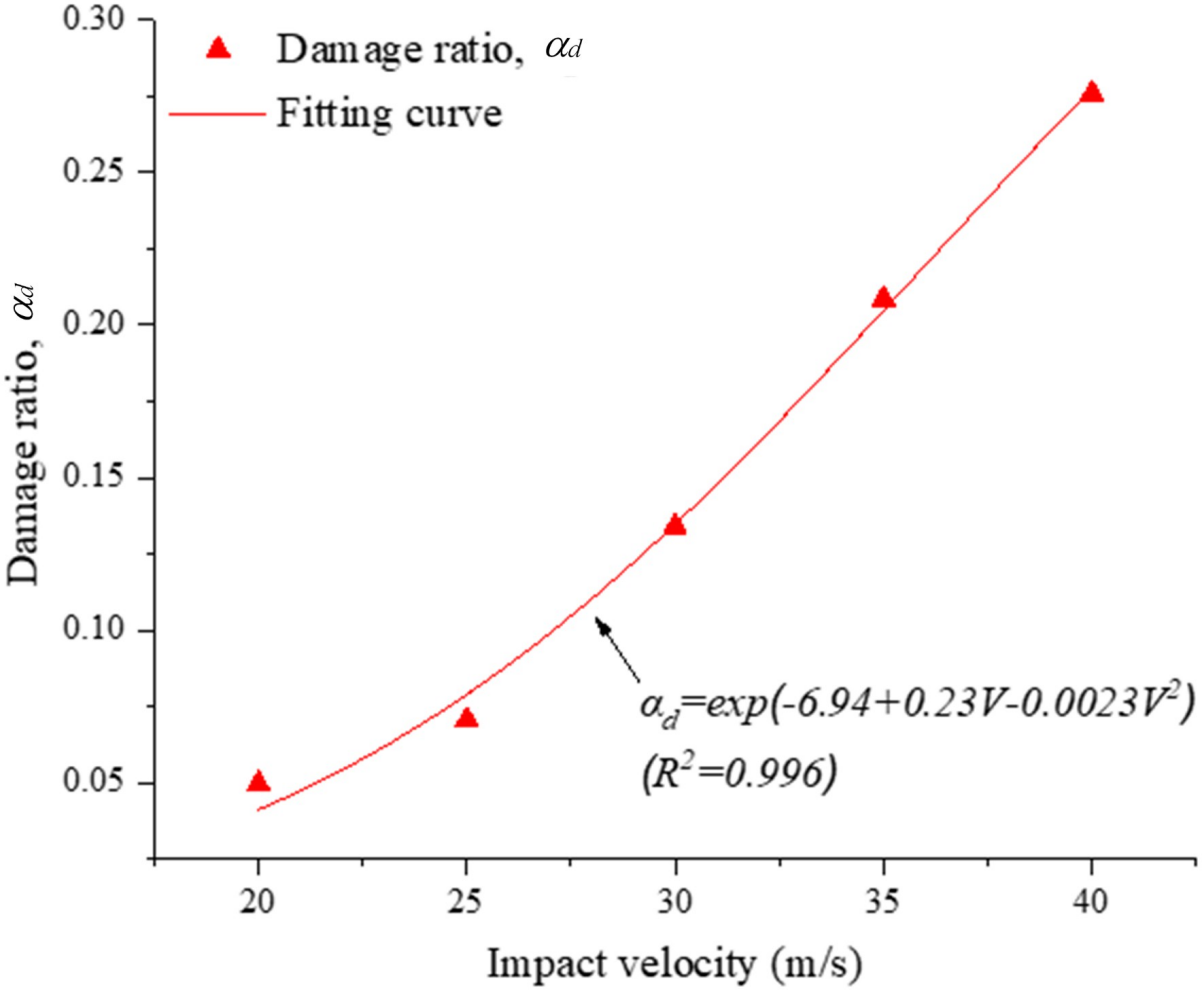
Variation in damage ratio (*α*_*d*_) with the impact velocity.

Fig 15 shows the typical evolution of the damage ratio during the impact with time under different impact velocities. As the impact velocity gradually increases, the increasing trend of the damage ratio becomes sharper. Also, when the impact velocity is 20.0 m/s, an obvious step-shaped curve appears during impact. In addition, as the impact velocity increases to 30.0 m/s, the damage ratio still slightly increases after 0.25 ms, and this phenomenon becomes stronger when the impact velocity increases to 40.0 m/s, which shows that the fragment number still slightly increases after 0.25 ms when the impact velocity exceeds 3.0 m/s. Fig 16 shows the relationship between the damage ratio and impact velocity, where the damage ratio during impact increases with the impact velocity via an exponential relationship. As the impact velocity increases from 20.0 to 40.0 m/s, the corresponding damage ratio increases progressively from 0.05 to 0.28.

### 4.3 Fragmentation and size distribution

**Fig 17:**
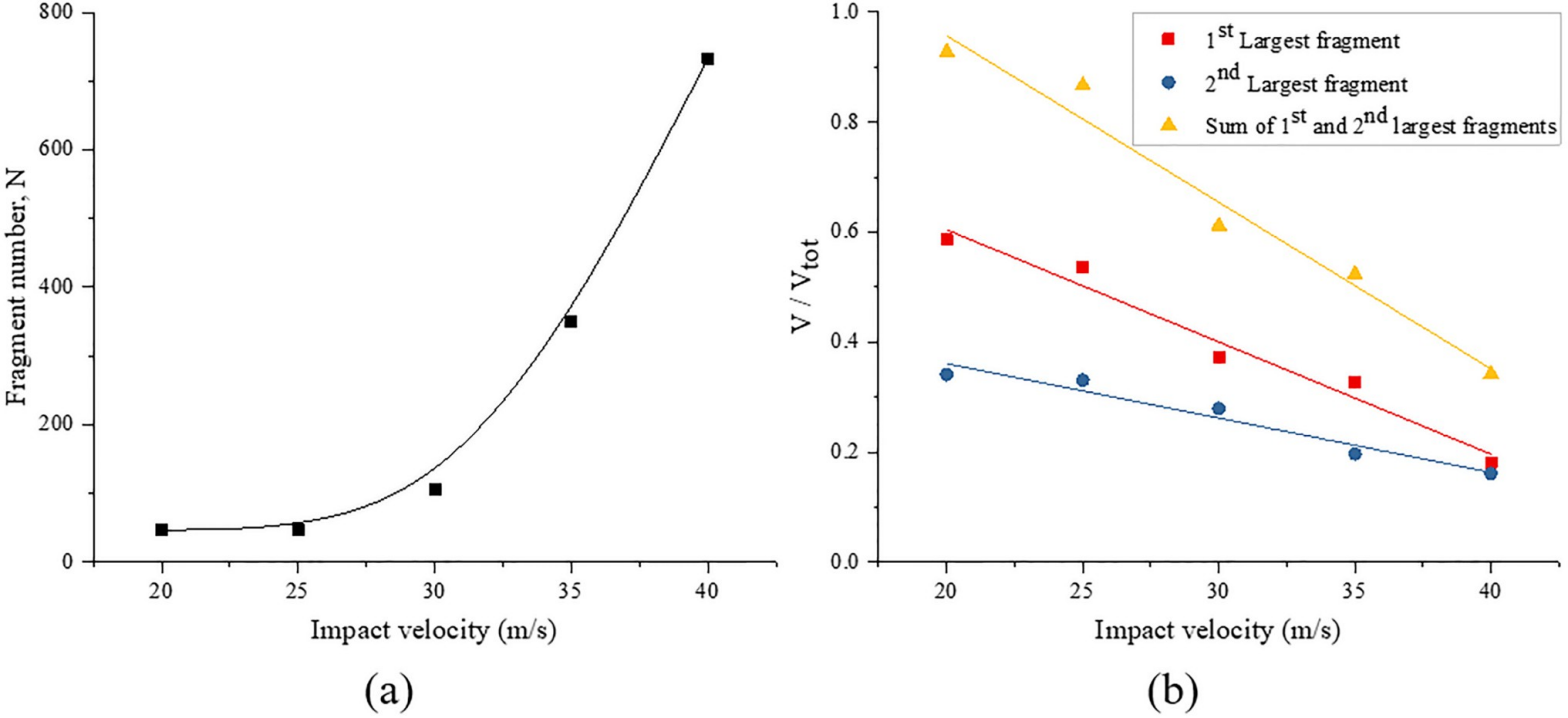
Fragmentation characteristics of the sample under different impact velocities. (a) fragment number; (b) the sizes of the two largest fragments and their sum.

The fragmentation and size distribution under different impact velocities are analyzed in this section. Fig 17 shows the fragmentation characteristics of the sample under different impact velocities. Fig 17a shows that the number of fragments gradually increases with increasing impact velocity. In particular, as the impact velocity increases from 20.0 m/s to 30.0 m/s, the fragment number only slightly increases (from 47 to 106). However, when the impact velocity exceeds 30.0 m/s, the fragment number increases sharply (from 106 to 732). Fig 17b shows the volumes of the two largest fragments, *V*_*1st*_ and *V*_*2nd*_, as well as their combined volume, *V*_*12*_,normalized by the initial volume of the sample, *V*_*tot*_, under different impact velocities. The volumes of the two largest fragments and their sum both decrease with increasing impact velocity. Also, as the impact velocity gradually increases, the volume difference between the two largest fragments decreases, especially when the impact velocity is 40.0 m/s, and the volumes of the two largest fragments are similar. As the impact velocity increases from 20.0 m/s to 40.0 m/s, the volumes of the two largest fragments gradually decrease from 58.6% and 34.1% to 18.1% and 16.1%, respectively. The combined volume of the two largest fragments gradually decreases from 92.8% to 34.2%, and other fragments are effectively pulverized.

To analyze the fragment size distribution, we defined the characteristic fragment size as follows [17]:

$$d=\sqrt[{3}]{V_{f}/V_{tot}}$$ (12)

where *V*_*f*_ is the volume of the fragment, which is calculated as the total volume of solid elements in the fragment; and *V*_*tot*_ is the initial volume of the rock block.

**Fig 18:**
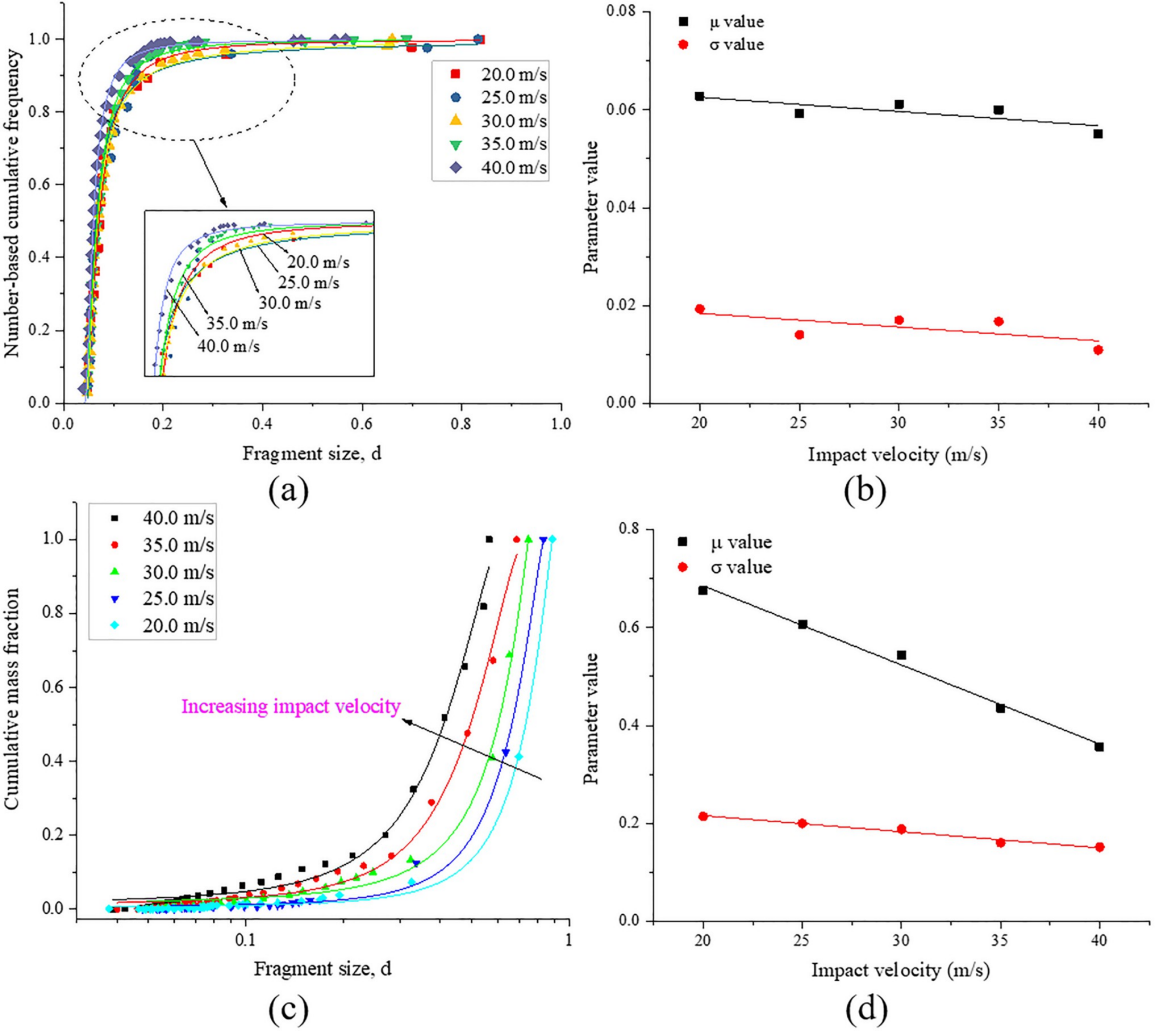
Cumulative size distribution of fragments based on (a) mass and (c) number (the solid lines are fitted curves using the generalized extreme value distribution); (b) and (d) show the corresponding fitting parameters *μ* and *σ*.

Regarding the fragment size distribution, many scholars have proposed various distribution functions, among which the most popular function is the Weibull distribution. However, Hogan et al. [24] proposed a three-parameter generalized extreme value distribution to analyze the fragment size distribution and showed that this distribution function can fit experimental data more accurately than the Weibull distribution. The three-parameter generalized extreme value distribution can be described as follows:

$$F(d;\mu ;\sigma ;\xi )=\text{exp}\left\{-{\left[1+\xi \left(\frac{d-\mu }{\sigma }\right)\right]}^{-1/\xi }\right\}$$ (13)

where *μ*, *σ* and *ξ* are the location, scale, and shape parameters, respectively. The shape parameter *ξ* is used to control the shape of the fitting curves, and the mass-based and number-based distribution curves show a different shape when *ξ* < 0 or *ξ* > 0. Due to a lack of physical meaning, this parameter is not analyzed in detail in this study. Fig 18 shows the typical distributions of the fragments obtained from the simulation results. Fig 18a and 18c shows that the distribution based on the mass and number of fragments can be fitted with good accuracy using the generalized extreme value distribution.

In the generalized extreme value distribution, the parameter *μ* is determined by the average size of the rock fragments, and *σ* determines the range of the fragment size distribution. Therefore, the study of two parameters can describe the influence of the impact velocity on the fragment size distribution. As shown in Fig 18b, the two parameters slightly decrease with increasing impact velocity for the fragment size distribution weighted by the fragment number. Fig 18d shows the fragment size distribution weighted by fragment mass, and indicates that the two parameters decrease linearly with increasing impact velocity. This result shows that as the impact velocity increases, the average fragment size gradually decreases, and the corresponding fragment size distribution range narrows. This behavior is expected because the increasing impact velocity generally leads to a reduced fragment size. The fragmentation intensity and fragment number also increase with increasing impact velocity.

### 4.4 Distribution of the fragment flying velocity and angle

**Fig 19:**
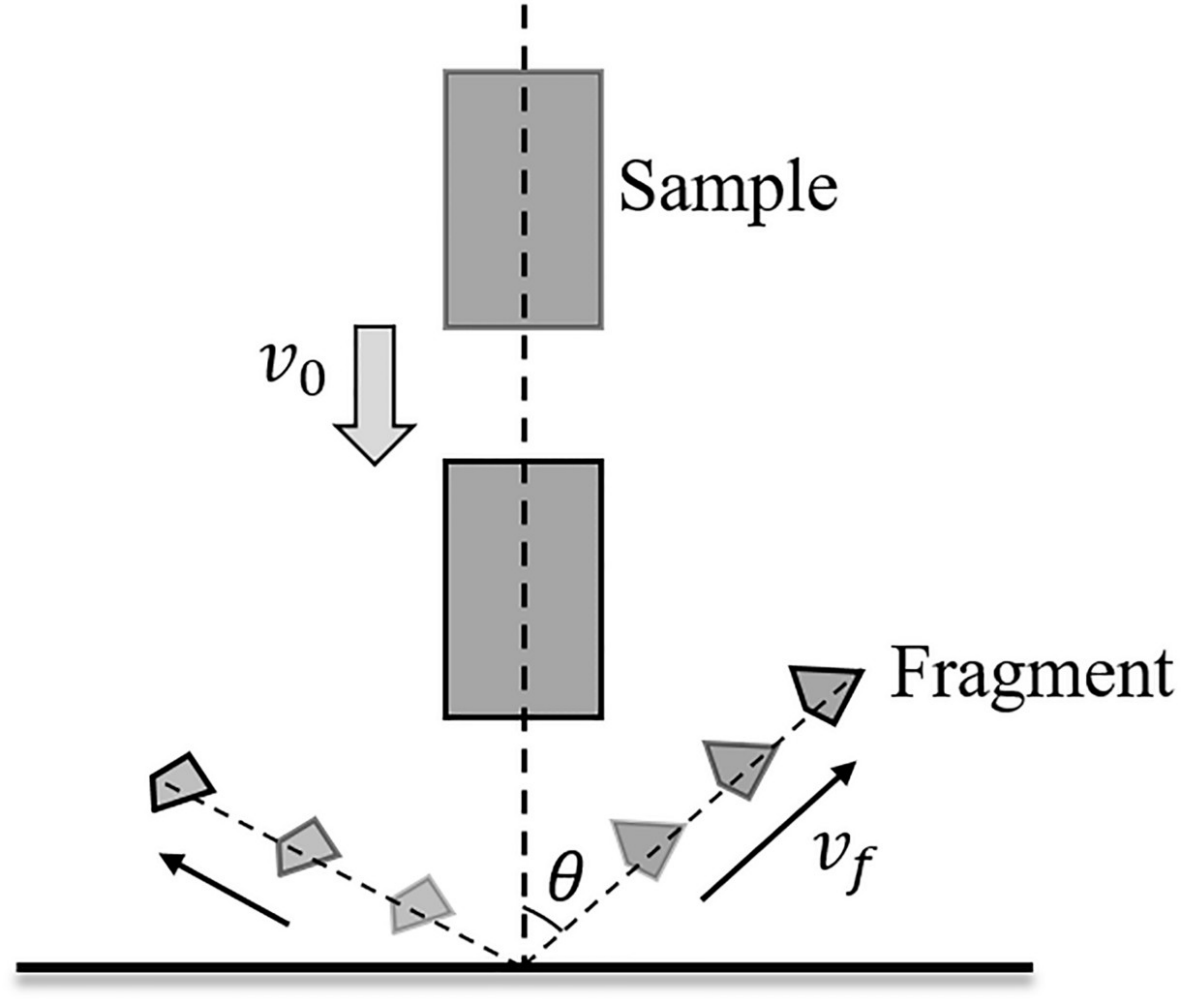
Schematic diagram of fragment flying velocity and angle.

**Fig 20:**
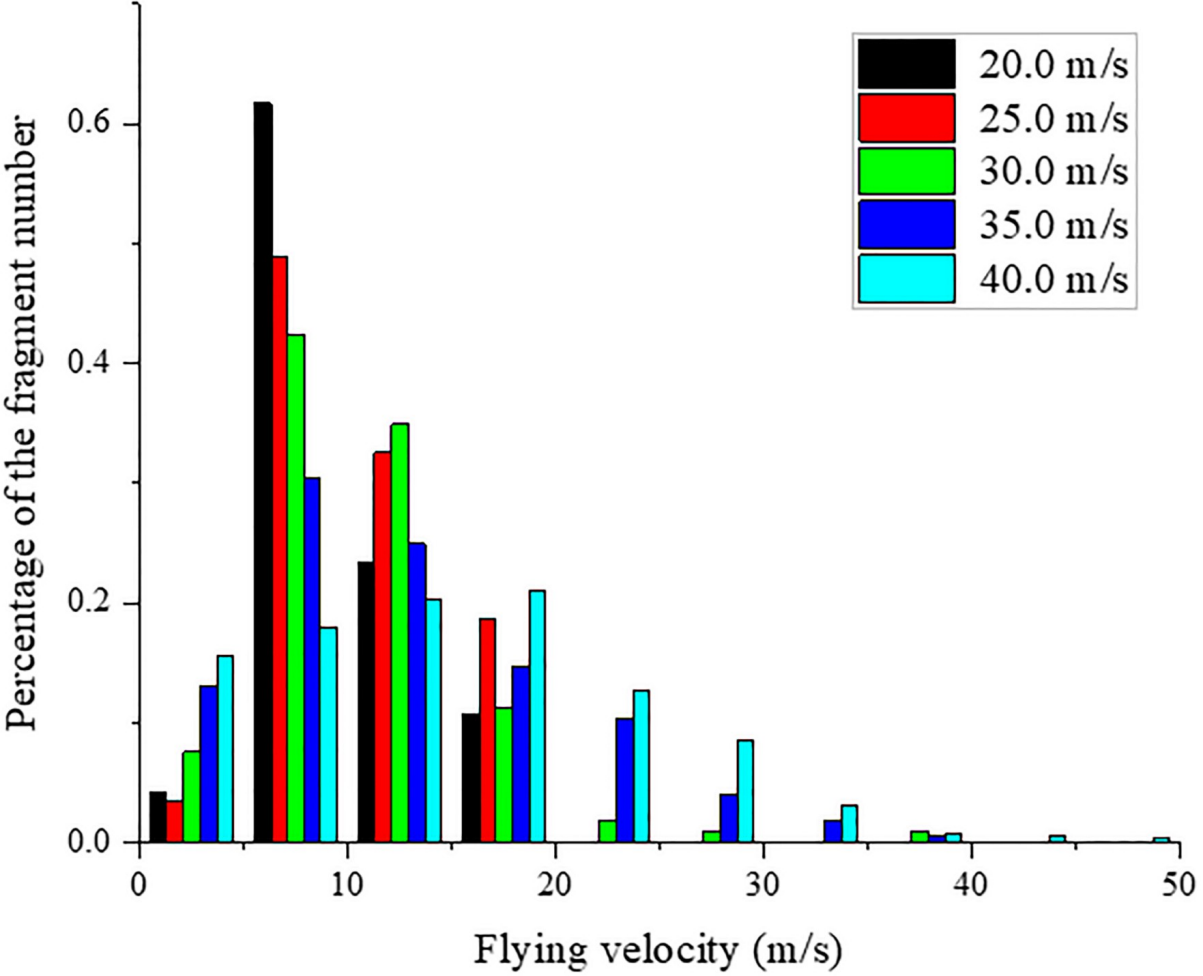
Distribution of fragment flying velocity at different impact velocities.

The fragment flying velocity (*v*_*f*_) and angle (*θ*) are important parameters in rock dynamic fragmentation, and their schematic diagram is shown in Fig 19. The fragment flying velocity and angle distribution are analyzed in this section. Fig 20 shows the distribution of fragment flying velocity at different impact velocities. The abscissa indicates the velocity interval of the fragment, and the ordinate indicates the percentage of the fragment number. Because the fragmentation intensity and fragment number are weak under impact velocities of 20.0 and 25.0 m/s, the flying velocities of most fragments are in the ranges of 5.04–16.45 and 5.56–18.05 m/s, respectively. When the impact velocity increases to 30.0 m/s, the fragmentation intensity and fragment number increase markedly, and the sample begins to produce many fragments that are generated by squeezing and splitting. Therefore, the fragment flying velocity increases, and the maximum flying velocity is approximately 38.31 m/s. Most fragments are launched at a velocity between 5.19–19.68 m/s. When the impact velocity increases to 35.0 m/s, the fragmentation intensity and fragment number increase, and the fragment flying velocity is mostly concentrated in the range of 5.01–24.88 m/s. The maximum flying velocity is approximately 41.05 m/s. When the impact velocity increases to 40.0 m/s, the fragmentation intensity and fragment number increase markedly; however, the distribution difference in each interval is relatively small when the fragment flying velocity is lower than 30.0 m/s. The maximum flying velocity is approximately 51.52 m/s. Fig 20 thus indicates that the fragment flying velocity gradually increases with increasing impact velocity. When shear failure is generated on the rock sample, the fragment flying velocity is always less than the impact velocity; however, squeeze and split failure occurs on the rock sample when the impact velocity is higher than 30.0 m/s, the fragment flying velocity starts to be greater than the impact velocity, and the number of fragments increases with increasing impact velocity.

**Fig 21:**
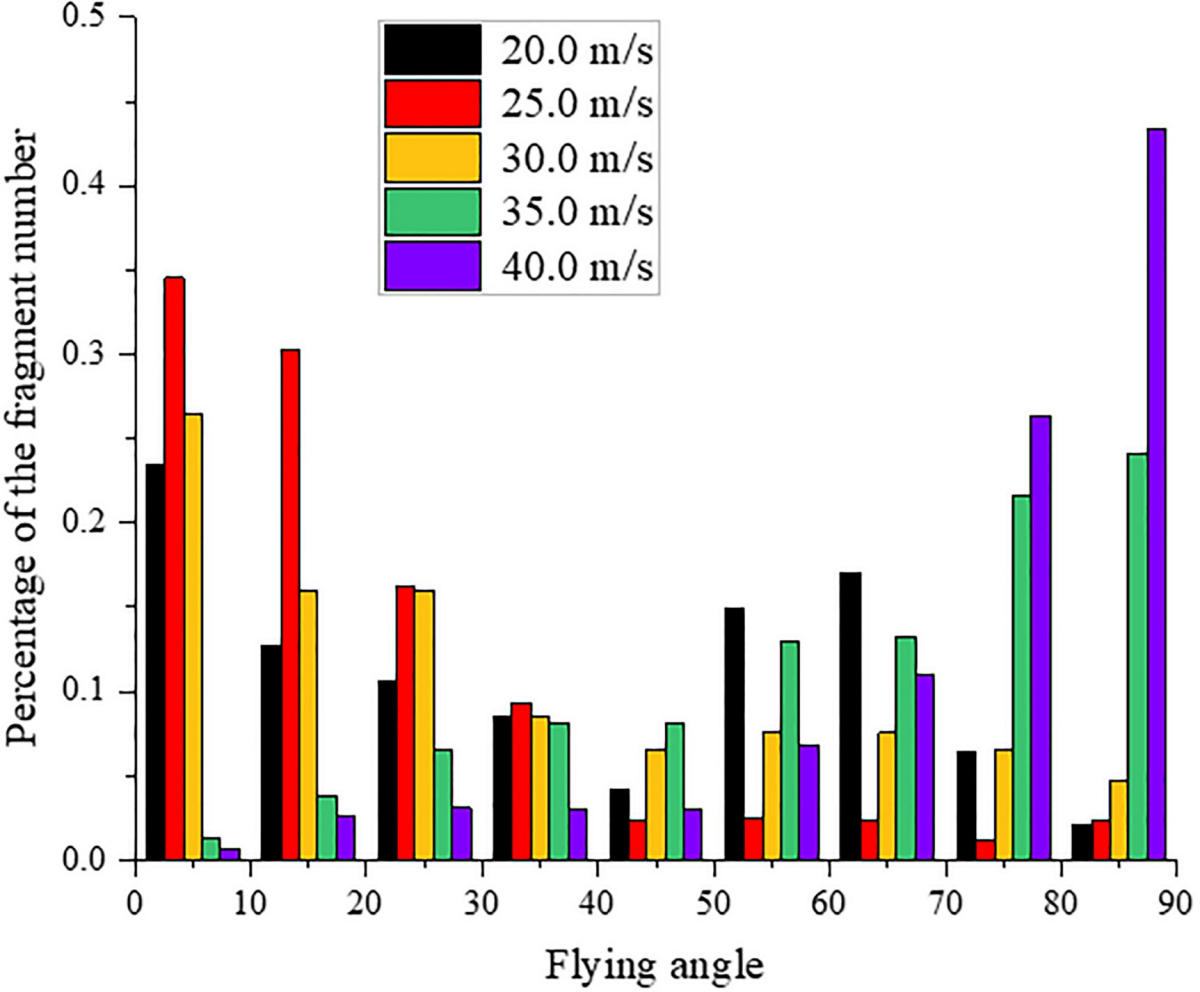
Distribution of the fragment flying angle at different impact velocities.

Similarly, the distribution of the fragment flying angle is shown in Fig 21. The flying angle is calculated by the angle between the direction of the fragment flying velocity and the direction opposite to the initial impact direction of the sample, as shown in Fig 19. When the impact velocity is 20.0 m/s, approximately 60% of the fragment has a flying angle between 0° and 45°, and approximately 32% of the fragment is launched with an angle between 45° and 90°. The reason for this result may be that shear failure occurred in the part of the sample near the impact face. Some fragments bounced back directly after impacting the dam-board, while a small number of fragments near the impacting face were launched at an angle greater than 45° due to extrusion. When the impact velocity is 25.0 m/s, approximately 93% of the fragment has a flying angle between 0° and 45° because the sample is sheared and broken into two large fragments, and most fragments bounce back after impacting the dam board. When the impact velocity increases to 30.0 m/s, approximately 74% of the fragment is launched with an angle between 0° and 45°, and the distribution of the angle between 45° and 90° begins to increase by approximately 26%. When the impact velocity increases to 35.0 m/s, the distribution of the fragment flying angle between 45° and 90° increases to 72%, and approximately 28% of the fragment has a flying angle between 0° and 45°. When the impact velocity is 40.0 m/s, most fragments (approximately 88%) are launched at an angle between 45° and 90°. The remaining fragments are nearly evenly distributed at other angles. When the impact velocity is greater than 30 m/s, the distribution of the fragment flying angle between 45° and 90° gradually increases with increasing impact velocity. The reason for this result may be that the part near impacting face is squeezed to a large number of fragments and the part of the sample that is far away from the impacting face is directly split into several large fragments due to the increase of impact force, resulting in the gradual decrease in the number of fragment directly rebounding after impacting the dam-board, while the number of fragment with a launch angle between 45° and 90° gradually increases.

## 5. Conclusions

In this study, laboratory impact tests on rock samples with different impact velocities were first conducted using a newly developed gas-driven rock impact apparatus. The fragmentation process and strain localization of rock samples under different impact velocities were analyzed. Then, a coupled FDEM model was developed using Abaqus software, based on which the progressive fracture process; evolution of energy and damage; fragmentation characteristics; and fragment flying characteristics at different impact velocities were investigated. The following conclusions can be drawn:

1. When the impact velocity increases from 20.0 to 30.0 m/s, the fragmentation intensity continues to increase. With increasing impact velocity, the failure pattern of the rock sample gradually changes from shear failure to splitting failure, and the angle of the shear failure plane gradually decreases as the location slowly moves away from the impacting face. The 3D-DIC technique can accurately predict the crack propagation direction through changes in the strain field. As the impact velocity increases, the strain localization area gradually increases, and the strain localization in the axial and vertical directions becomes increasingly obvious.
2. As the impact velocity increases, the broken zone gradually deviates away from the impacting face, and the number of cracks increases significantly. Numerical results indicated that the impact-induced fragmentation gradually changes from shear failure to squeeze (near the impacting face) and tensile (away from the impacting face) failure with increasing impact velocity. As the impact velocity increases from 20.0 to 40.0 m/s, the normalized dissipated energy gradually increases from 0.74 to a critical value 0.88. Correspondingly, the corresponding damage ratio increases progressively from 0.05 to 0.28.
3. The number and volume of fragments are markedly affected by the impact velocity. When the impact velocity increases from 20 m/s to 30 m/s, the number of fragments slowly increases from 47 to 106. When the impact velocity increases from 30 m/s to 40 m/s, the number of fragments increases sharply from 106 to 732. Meanwhile, the average volume of the fragments will decrease as the impact velocity increases. When the impact velocity increases from 20 m/s to 40 m/s, the combined volume of the two largest fragments gradually decreases from 92.8% to 34.2%.
4. As the impact velocity increases, the average fragment flying velocity gradually increases. Regardless of the impact velocity, the majority of the fragment flying velocities are within a range of 5.0–25.0 m/s. When the impact velocity increases to 30.0 m/s, the largest flying velocity starts to be higher than the impact velocity. Similarly, the fragment flying angle gradually increases with increasing impact velocity. When the impact velocity is lower than 30.0 m/s, most fragments are launched at an angle between 0°-45°. The flying angles of most fragments are in the range of 45°-90° when the impact velocity is greater than 30.0 m/s.
